# Four Undescribed Pyranones from the *Scutellaria formosana*-Derived Endophytic Fungi *Ascomycota* sp. FAE17

**DOI:** 10.3390/molecules28145388

**Published:** 2023-07-13

**Authors:** Jianni Yang, Yang Hui, Zhaoxia Chen, Guangying Chen, Xiaoping Song, Zhenfan Sun, Changri Han, Wenhao Chen

**Affiliations:** 1 Key Laboratory of Tropical Medicinal Resource Chemistry of Ministry of Education, College of Chemistry and Chemical Engineering, Hainan Normal University, Haikou 571158, China; jianni1216@163.com (J.Y.); 070066@hainnu.edu.cn (Y.H.); 17689849894@163.com (Z.C.); chgying123@163.com (G.C.);; 2 Key Laboratory of Tropical Medicinal Plant Chemistry of Hainan Province, Haikou 571158, China; 3 Key Laboratory of Medicinal and Edible Plants Resources of Hainan Province, Hainan Vocational University of Science and Technology, Haikou 571158, China

**Keywords:** *Scutellaria formosa*, *Ascomycota* sp. FAE17, secondary metabolites, biological activity

## Abstract

Four undescribed pyranone derivatives, named ascomycopyrones A–D (**1**–**4**), as well as one known analogue simplicilopyrone (**5**) (this is the first study to report the absolute configuration), were isolated from the endophytic fungus *Ascomycota* sp. FAE17 derived from the flowers of *Scutellaria formosa*. The structures of these pyranones were identified by comprehensive spectroscopic and MS analyses, and the absolute configurations were determined by their experimental and quantum chemical electronic circular dichroism (ECD) calculations. All isolated compounds were tested for various bioactivities, including antibacterial, cytotoxic activity, and NO inhibitory activity. Unfortunately, none of the compounds showed significant bioactivities.

## 1. Introduction

Endophytic fungi are a promising and important source of novel and bioactive natural products [1,2,3,4]. A range of bioactive specialized metabolites have been reported from plant endophytic fungi, including polycyclic beticolin, emestrin, isoindoline alkaloid xylarin A, diketopiperazine alkaloids phaeosphaones A–D, and ansamacrolactams catellatolactams A–C [5,6,7,8,9,10]. It has been suggested that plant endophytic fungi could produce bioactive compounds of plant origin in a sustainable way, thus indicating the need for further research in this context.

*Scutellaria formosa* belongs to the genus *Scutellaria* of the family Labiatae (Lamiaceae), which is a very rare perennial herb mainly distributed in Hainan, Guangdong, Yunnan, and other regions of China. It mostly grows in special areas with humid soil in the shade of forests at an altitude of 450–550 m in southern Hainan [11]. Previous phytochemical investigations of the plant led to the identification of several *neo*-clerodane diterpenoids and flavonoids, which revealed in vitro cytotoxicity against various cancer cell lines, anti-inflammatory properties, and inhibitory effects against HIV lytic replication [11,12,13,14]. However, information on the constituents and bioactivity of the secondary metabolites of endophytic fungus from *S. formosa* is lacking. So far, about 36 compounds have been isolated and identified from *Ascomycota* sp., which have been evaluated as antibacterial and anti-inflammatory, with significant inhibition effects against α-glucosidase and antioxidatives [15,16,17,18,19].

**Figure 1:**
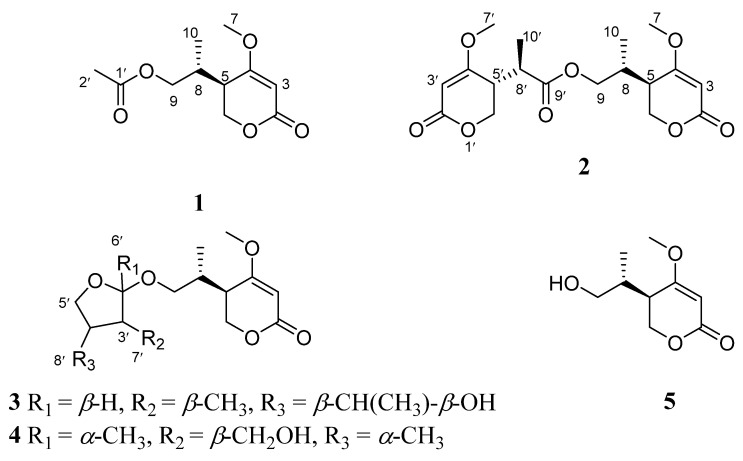
The structures of compounds **1**–**5**.

In the course of further development and utilization of the pharmaceutical plant source of *S. formosa*, our previous investigation on endophytic fungi from this plant resulted in the discovery of several types of new compounds [20]. In this study, we investigated the endophytic fungus *Ascomycota* sp. FAE17 which was isolated from the flowers of *S. formosa*, collected in Hainan Province. Purification of the EtOAc extract from the fermented rice culture afforded four previously undescribed pyrone compounds, named ascomycopyrones A–D (**1**–**4**), as well as one known compound (**5**) (Figure 1). Herein, we describe the isolation, structural elucidation, as well as the antibacterial, cytotoxic activity, and NO inhibitory activity of all the compounds.

## 2. Results and Discussion

**Figure 2:**
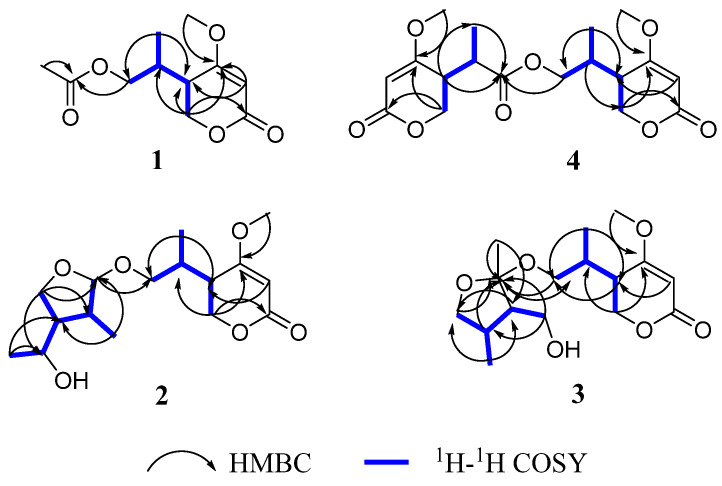
^1^H-^1^H COSY and key HMBC correlations for compounds **1**–**4**.

Compound **1** was obtained as a yellow oil. The positive HRESIMS of **1** showed a pseudomolecular ion peak at *m*/*z* 251.0877 [M + Na]^+^ and established a molecular formula of C_11_H_16_O_5_, which suggested four degrees of unsaturation. The ^1^H NMR (Table 1) and HSQC spectra showed the presence of one olefinic proton of a trisubstituted alkene [*δ*_H_ 5.22 (1H, s)], two sets of oxymethylene protons [*δ*_H_ 4.32 (2H, d, *J* = 3.6 Hz) and 3.96 (1H, dd, *J* = 11.2, 6.4 Hz, 3.91 (1H, dd, *J* = 11.2, 6.4 Hz)], one methoxy group [*δ*_H_ 3.73 (3H, s)], two methine protons [*δ*_H_ 2.49 (1H, m), 2.17 (1H, m)], and two methyl group [*δ*_H_ 2.00 (3H, s), 0.93 (3H, d, *J* = 7.2 Hz)]. The ^13^C NMR and DEPT spectra (Table 1) displayed signals for one typical carbonyl carbon of an *α*, *β*-unsaturated lactone moiety (*δ*_C_ 165.8), one plausible carboxylic carbon (*δ*_C_ 170.3), one oxygenated quaternary (*δ*_C_ 174.2), which could belong to enol olefin, three methine (*δ*_C_ 91.0, 38.7, 33.3), two methylene (*δ*_C_ 67.1, 66.4) carbons, one methoxy (*δ*_C_ 56.2), and two methyl (*δ*_C_ 20.6, 14.4) groups. The olefinic proton *δ*_H_ 5.22 was assigned as H-3 of the *α*, *β*-unsaturated moiety on the basis of its HMBC correlations (Figure 2) with C-5 (*δ*_C_ 38.7) and C-4 (*δ*_C_ 174.2). Furthermore, the ^1^H-^1^H COSY spectrum (Figure 2) showed the spin system of H-8/H-5/H-6 and H-9/H-8/H-10. This finding, together with the HMBC correlations of H-6 (*δ*_H_ 4.32) with C-2 (*δ*_C_ 91.0), C-4 (*δ*_C_ 174.2) and C-5 (*δ*_C_ 38.7), H-10 (*δ*_H_ 0.93) with C-5 (*δ*_C_ 38.7) and C-9 (*δ*_C_ 66.4), suggested the presence of a 5-substituted 4-methoxy-2*H*-pyran-2-one (*α*-pyrone) moiety [21]. The substituent at C-4 (*δ*_C_ 174.8) was a methoxy group according to a ^3^*J* HMBC correlation of the methoxy protons at *δ*_H_ 3.73 (3H, s) with this carbon. In addition, resonances for an acetyl unit [(*δ*_C_/*δ*_H_ 20.6/2.00 (3H, s), *δ*_C_ 170.3) were observed in the NMR spectra and an HMBC correlation from H-9 (*δ*_H_ 3.96, 3.91) to the carbonyl carbon at *δ*_C_ 170.3. Accordingly, the planar structure of **1** was determined as shown (Figure 1).

**Figure 3:**
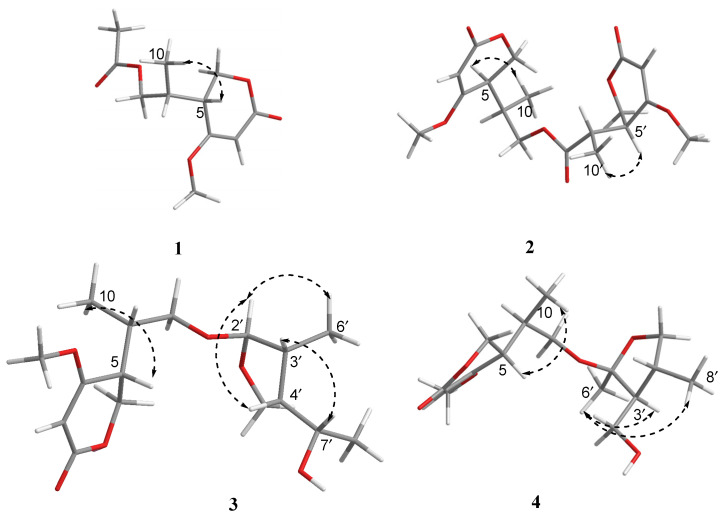
Key NOESY correlations for compounds **1**–**4**.

**Figure 4:**
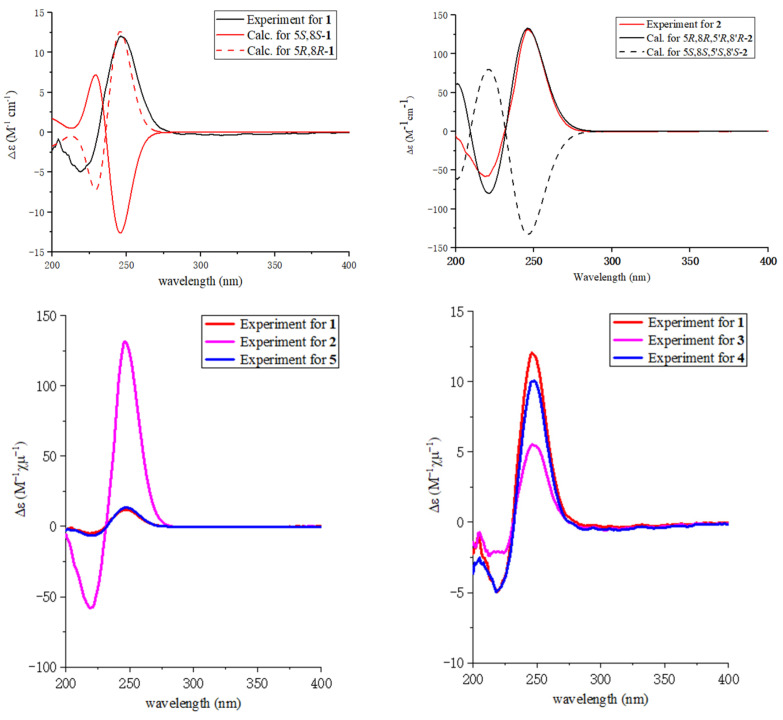
Experiment CD spectra and the calculated ECD spectra of compounds **1**–**5**.

The relative configuration of **1** was elucidated by analysis of NOESY data (Figure 3). In the NOESY spectrum, there was NOE correlation between H-5 and Me-10, which suggested the anti relationship between H-5 and H-8 [22,23]. Therefore, the relative configuration of **1** was determined to be 5R*, 8R*. In order to determine the absolute configuration of **1**, the theoretical electronic circular dichroism (ECD) spectra (Figure 4) of two possible stereoisomers of (5*R*, 8*R*)-**1** and its enantiomer were calculated using a time-dependent density functional theory (TDDFT) calculation. A computational modeling study was conducted by Gaussian 09 [24] and the ECD spectrum was simulated in SpecDis by overlapping Gaussian functions for each transition [25]. The calculated ECD curve of (5*R*, 8*R*)-**1** (Figure 4) was consistent with the experimental one. Thus, the absolute configuration of **1** was determined as 5*R*, 8*R* and named as ascomycopyrone A.

**Figure 5:**
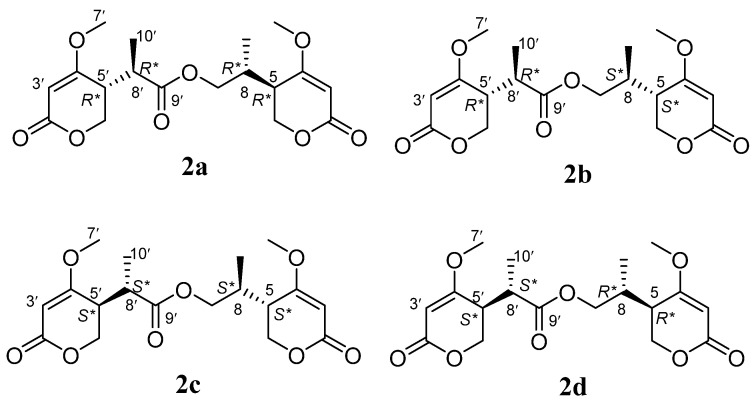
The four possible configurations of compound **2**.

**Figure 6:**
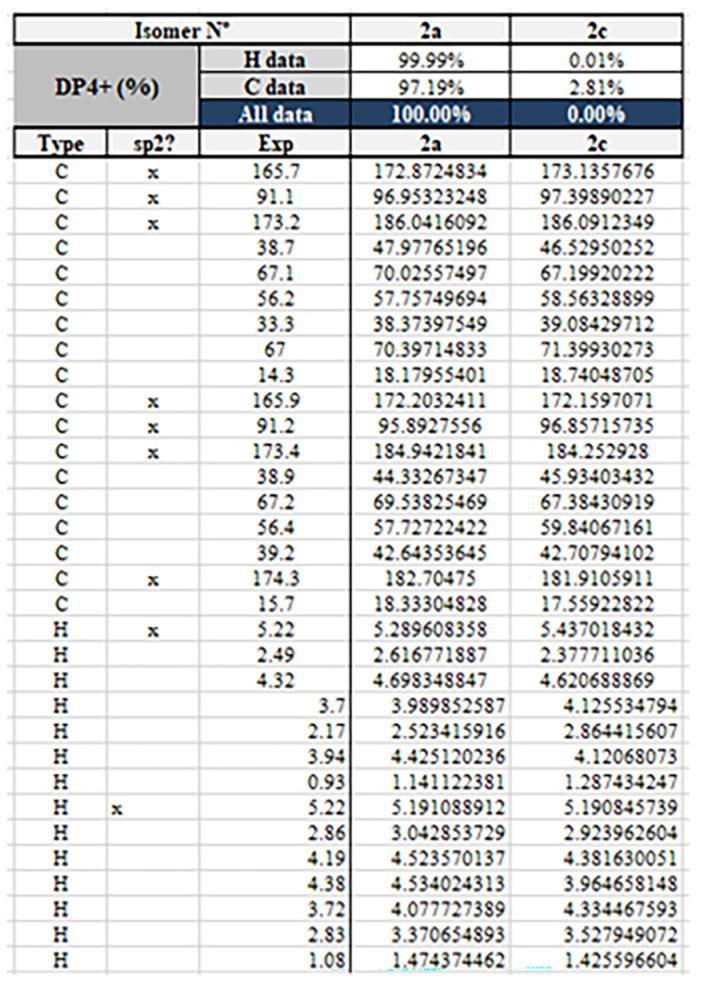
The DP4+ calculations for compound **2**.

Compound **2** was isolated as a yellow oil and had the molecular formula C_18_H_24_O_8_ as determined by the HRESIMS peak at *m*/*z* 391.1349 [M + Na]^+^. The ^1^H and ^13^C NMR data (Table 1) of **2** revealed structural features closely related to the known simplicilopyrone **5** [22]. The remaining resonances for **2** showed structural similarity to oxidation products of **5**, implying that **2** could be a heterodimer derived from **5**. The HMBC correlations (Figure 2) from H-10′ to C-9′, C-8′, from H-6′ to C-8′, and from H-9 to C-9′ established the C9′-C8′-C5′ linkage between the two monomers, thereby completing the gross structure of **2** as shown in Figure 1. The relative configuration of **2** was elucidated by analysis of its NOESY data (Figure 3). The key NOESY correlations of H-5/Me-10 and H-5′/Me-10′ indicated that H-5 and H-10, H-5′, and H-10′ were on the same face [22]. Thus, the four possible configurations (5R*, 8R*, 5′R*, 8′R* **2a,** and its enantiomer **2c**, 5S*, 8S*, 5′R*, 8′R* **2b,** and its enantiomer **2d**) of the stereogenic carbons were predicted. The DP4+ calculations for compound **2** were further performed, where both ^13^C and ^1^H data revealed **2a** possessed 100% probabilities in all calculations (Figure 5 and Figure 6). Finally, the ECD spectrum of **2a** and **2c** was obtained, indicating that the result was consistent with that observed from the DP4^+^ analysis (Figure 4). Hence, compound **2** was assigned to be 5*R*, 8*R*, 5′*R*, 8′*R*, and named as ascomycopyrone B.

Compound **3** was isolated as a colorless oil and gave a pseudomolecular ion [M + Na]^+^ peak at *m*/*z* 337.1608 by HRESIMS, consistent with a molecular formula of C_16_H_26_O_6_ (four degrees of unsaturation). Its ^1^H and ^13^C NMR spectrum data (Table 2) revealed the same 4-methoxy-2*H*-pyran-2-one moiety and 2-substituted 1-hydroxypropyl group as found in **1**. In addition, NMR resonances for two methyls [*δ*_C_/*δ*_H_ 22.6/1.08 (3H, d, *J* = 6.4 Hz), 18.0/0.96 (3H, d, *J* = 7.2 Hz)], one oxygenated methylene [*δ*_C_/*δ*_H_ 69.2/3.90 (1H, t, *J* = 8.8 Hz)/3.66 (1H, t, *J* = 8.8 Hz)], two methines [*δ*_C_/*δ*_H_ 53.9/1.59 (1H, qd, *J* = 8.0, 6.0 Hz), 42.0/.80 (1H, td, *J* = 6.8, 2.4 Hz)], two oxygenated methines [*δ*_C_/*δ*_H_ 110.8/4.58 (1H, d, *J* = 2.4 Hz), 67.4/3.54 (1H, t, *J* = 6.8 Hz)] were observed in the spectra of **3**. The other partial structural fragments were determined by 2D NMR data, including COSY, HSQC, and HMBC experiments. From the ^1^H-^1^H COSY spectrum (Figure 2), it was possible to establish the proton sequence from H-2′ (*δ*_H_ 4.58) to H-6′ (*δ*_H_ 0.97) to H-4′ (*δ*_H_ 1.59) through H-3′ (*δ*_H_ 1.80); H-5′ (*δ*_H_ 3.90/3.66) to H-7′ (*δ*_H_ 3.54) through H-4′ (*δ*_H_1.59); and H-7′ (*δ*_H_ 3.54) to H-8′ (*δ*_H_ 1.07). The presence of 3′-methyl-4′-ethoxy furan moiety was determined on the basis of the key HMBC correlations (Figure 2) from H-6′ (*δ*_H_ 0.97) to C-2′ (*δ*_C_ 110.8), C-3′ (*δ*_C_ 42.0) and C-4′ (*δ*_C_ 53.9), H-8′ (*δ*_H_ 1.07) to C-7′ (*δ*_C_ 67.4) and C-4′ (*δ*_C_ 53.9). The above HMBC correlations also confirmed the linkages established by the COSY experiment. Moreover, the location of the moiety was positioned at C-9 through the ether bond by the crucial HMBC correlation from H-2′ (*δ*_H_ 4.58) to C-9 (*δ*_C_ 69.8). From these data, the planar structure of **3** was proposed. The relative configuration of **3** was elucidated from NOESY data (Figure 3). A NOESY correlation between H-5 (*δ*_H_ 2.44) and H-10 (*δ*_H_ 0.89) placed these protons on the same face. The relative configurations of H-2′, 3′, 4′, 7′ in the furan moiety was elucidated to be *trans* in H-2′ and H-4′, *cis* in H-3′, H-4′, and H-7′ on the basis of the NOESY correlations of H-6′/H-2′/H-4′and H-3′/H-7′. The absolute configuration of C-5, 8 was determined to be *R*, *R,* consistent with the CD spectrum (Figure 4) of **3**, in which the Cotton effect was observed at 200–275 nm, similarly to the case of compound **1**. Therefore, the structure of ascomycopyrone C (**3**) was thus determined.

Compound **4** was obtained as a colorless oil. The HRESIMS of **4** exhibited a [M + Na]^+^ peak at *m*/*z* 337.1604 and established a molecular formula of C_16_H_26_O_6_, implying four degrees of unsaturation. Comparison of the NMR spectroscopic data (Table 2) with those obtained for **3** showed that **4** was also a 4-methoxy-2*H*-pyran-2-one analogue of **3**. This significant difference could be explained by the formation of the 2′,3′,4′-substituent in **4**. This explanation was further supported by the COSY correlation (Figure 2) between H-7′, H-3′, and H-4′, H-5′, H-4′, and H-8′, and the HMBC correlations (Figure 2) from H-5′ to C-2′, H-8′ to C-5′ and C-3′, H-7′ to C-2′, and C-4′, H-6′ to C-2′, which led to a 2′,4′-dimethyl-4′-methylol furan moiety. In the NOESY spectrum (Figure 3), the NOE correlation from H-5 to Me-10 was observed, which suggested that the configuration should be 5R*, 8R*. The configurations of H-2′, H-3′, and H-4′ were determined as *cis* by the NOESY correlations of H-3′/H-6′ and H-8′/H-6′. The absolute configurations of C-5 and C-8 were proposed as 5*R* and 8*R* on the basis of CD data, which were in good agreement with the CD data (Figure 4) of compound **4**. Consequently, the structure of **4** was deduced and named as ascomycopyrone D.

The known compound was identified as simplicilopyrone (**5**) (Figure 1) by comparing the spectroscopic data with that reported in the literature [22]. Its absolute configuration was determined for the first time to be 5*R*, 8*R*, in which the Cotton effect was observed at 200–275 nm, consistent with the CD spectrum of compound **1** (Figure 4). Compounds **1**–**5** all belong to pyranones, which contain a 5-substituted 4-methoxy-2*H*-pyran-2-one skeleton. So far, only four compounds have been isolated from fungus *Simpilcillium* sp. and myxobacteria *Nannocystis pusilla* [22,26]. This study would enrich the structural diversity of this class compounds.

**Figure 7:**
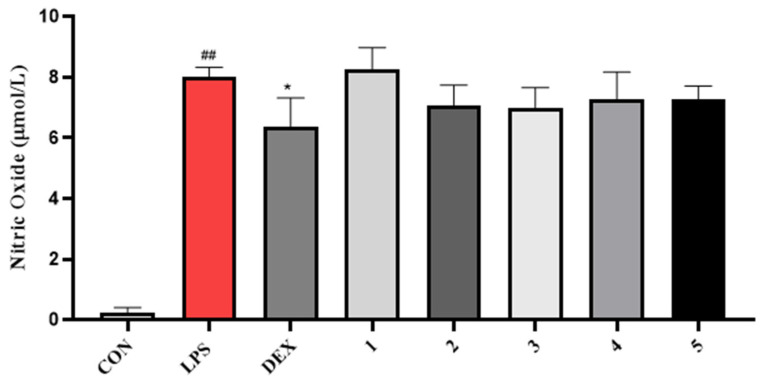
Impact of compounds **1**–**5** on NO production in LPS-treated RAW 264.7 cells. ^##^ *p* < 0.01 vs. Con, * *p* < 0.01 vs. LPS. *n* = 6.

Compounds **1**–**5** did not exhibit good antibacterial activity against *Escherichia coli*, *Staphylococcus aureus*, *Enterococcus faecalis*, and *Stenotrophomonas maltophilia*, with MIC values over 64 μg/mL (Table 3). In general, compounds **3** and **5** showed better cytotoxic activity against MCF-7, A549, and Hela cell lines than **1**, **2**, and **4**. The hydroxyl group at **5** was more important than the ester substitution at **1** and **2** against MCF-7 and Hela cells. It was also found that the cytotoxic activity against MCF-7, A549, and Hela cells was obviously reduced by the presence of the 2′,4′-dimethyl-4′-methylol furan moiety as in **4**. Unfortunately, none of them showed significant activities while compared with the positive control adriamycin at the tested concentration of 100 µM (Table 4). All compounds **1**–**5** were tested for their NO inhibitory activity. Unfortunately, none of them showed significant inhibitory activity against NO production. The acetyl group at **1** seem to significantly decrease the inhibitory activity at the concentration of 50 µM (Figure 7).

## 3. Materials and Methods

### 3.1. General Experimental Procedures

Optical rotations were acquired by an Anton paar MCP 5100 modular circular polarimeter (JASCO, Tokyo, Japan). CD spectra and UV spectra were obtained from a Boilogic Mos-500 spectrometer (JASCO, Tokyo, Japan). The 1D and 2D NMR (COSY, HSQC, and HMBC) spectra were recorded on a Bruker AV spectrometer (400 MHz for ^1^H and 100 MHz for ^13^C, (Bruker Corporation, Switzerland) instrument using DMSO-*d*_6_ as a solvent. Tetramethyl silane (TMS) was used as an internal standard. HR-ESI-MS spectra were made on a Bruker APEX II spectrometer (Billerica, MA, USA). Semi preparative HPLC was carried out with Agilent 1260 prep-HPLC system, using Agilent Eclipse XDB-C18 (9.4 × 250 mm, 5 μm, Agilent Corporation, Santa Clara, CA, USA), respectively. Silica gel (200–300 mesh, 300–400 mesh Qingdao Marine Chemical Factory, Qingdao, China) and octadecylsilyl silica gel (YMC; 12–50 μm) were used for column chromatography (CC). Precoated silica gel plates (GF-254, Qingdao Marine Chemical Factory, Qingdao, China) were used for thin layer chromatography (TLC). All solvents used for extractions and chromatographic separations were of analytical grade and purchased from Xilong Chemical Reagent Factory (Guangzhou, China), with the exception of HPLC grade solvents used for HPLC separations.

### 3.2. Fungal Materials

The endophytic fungal strain FAE17 was isolated from the flowers of fresh *Scutellaria formosana* (Lamiacea) harvested from Hainan Bawangling National Nature Reserve of Hainan Province, China, in July 2020. *S. formosana* was identified by Prof. Qiong-Xin Zhong, College of Life Science, Hainan Normal University. The strain was identified based on morphological, physiological, and biochemical characteristics and sequencing of the DNA of the ITS region of the rRNA gene. The sequenced data of strain FAE17 were most similar (99%) to the sequence of *Ascomycota* sp., which had been submitted to GenBank with accession no. OR072796. The endophytic fungus *Ascomycota* sp. FAE17 had been preserved at the Key Laboratory of Tropical Medicinal Resource Chemistry of Ministry of Education, College of Chemistry and Chemical Engineering, Hainan Normal University, Haikou, Hainan, China.

### 3.3. Fermentation, Extraction, and Isolation

This strain of *Ascomycota* sp. FAE17 was cultured on potato dextrose agar medium at 28 °C. After 10 days, the agar plugs were cut into small pieces to incubate on solid rice medium in 1000 mL Erlenmeyer flasks (90 g rice and 120 mL water for each Erlenmeyer flask, the total weight of rice was 27 kg) to culture for further 60 days at 28 °C [20]. The fermented cultures of *Ascomycota* sp. FAE17 were extracted 3 times with EtOAc, and the solvents were evaporated under reduced pressure to obtain an extract (310.0 g).

The total crude extract was subjected to silica gel column chromatography (CC) eluted with petroleum ether/EtOAc (9:1–0:1, *v*/*v*) and EtOAc/CH_3_OH (9:1-1:1, *v*/*v*) to give 5 fractions (Fr.1–Fr.5). Fr.3 (30.7 g) was further separated by CC over reversed phase C18 silica gel using CH_3_OH/H_2_O as eluent (1:9, 2:8, 3:7,4:6, 6:4, 8:2, 10:0, *v*/*v*) to give 7 subfractions (Fr.3.1–Fr.3.7). Fr.3.1 (3.2 g) was rechromatographed on a silica gel column eluted with petroleum ether/EtOAc (9:1–3:7, *v/v*) to obtain 4 subfractions (Fr.3.1.1–Fr.3.1.4). Fr.3.1.1 (259.5 mg) was purified by semipreparative HPLC using an Agilent Eclipse XDB-C_18_ (250 × 9.4 mm, 5 μm) with CH_3_CN/H_2_O (8:92, *v*/*v*) to yield compounds **1** (40.6 mg), **3** (2.3 mg), and **4** (3.5 mg). The Fr.3.2 (7.6 g) was separated by CC over reversed phase C18 silica gel using CH_3_OH/H_2_O as eluent (1:9, 2:8, 3:7,4:6, 6:4, 8:2, 10:0, *v*/*v*) to obtain 8 subfractions (Fr.3.2.1–Fr.3.2.8). Fr.3.2.3 (302.6 mg) was subjected to semipreparative HPLC with CH_3_CN/H_2_O (15:85, *v*/*v*) to yield compound **2** (9.5 mg). Fr.3.2.4 (231.9 mg) was further purified by semipreparative HPLC with CH_3_CN/H_2_O (13:87, *v*/*v*) to obtain compound **5** (12.3 mg).

Ascomycopyrone A (**1**): pale yellow oil; [α]${]}_{D}^{20}$ = +529.3 (*c* 0.0061, MeOH); UV (MeOH) *λ*_max_ [log *ε*/(L·mol^−1^·cm^−1^)]: 249 (2.30) nm; IR (KBr) ν_max_: 1710 cm^−1^, 1622 cm^−1^; HR-ESI-MS *m*/*z* 251.0877 [M + Na]^+^ (calcd for C_11_H_16_O_5_Na, 251.0890). ^1^H and ^13^C NMR data are in Table 1.

Ascomycopyrone B (**2**): yellow oil; [α${]}_{D}^{20}$ = −98.1 (*c* 0.0020, MeOH); UV (CH_3_OH) *λ*_max_ [log *ε*/(L·mol^−1^·cm^−1^)]: 249 (2.66), 300 (0.43) nm; IR (KBr) ν_max_: 1710 cm^−1^, 1621 cm^−1^; HR-ESI-MS *m*/*z* 391.1349 [M + Na]^+^ (calcd for C_18_H_24_O_8_Na, 391.1349). ^1^H and ^13^C NMR data are in Table 1.

Ascomycopyrone C (**3**): colorless oil; [α${]}_{D}^{20}$ = −102.2 (*c* 0.0014, MeOH); UV (MeOH) *λ*_max_ [log *ε*/(L·mol^−1^·cm^−1^)]: 241 (2.45) nm; IR (KBr) ν_max_: 3446 cm^−1^, 1622 cm^−1^; HR-ESI-MS *m*/*z* 337.1608 [M + Na]^+^ (calcd for C_16_H_26_O_6_Na, 337.1622). ^1^H and ^13^C NMR data are in Table 2.

Ascomycopyrone D (**4**): colorless oil; [α${]}_{D}^{20}$ = +80.4 (*c* 0.0015, MeOH); UV (MeOH) *λ*_max_ [log *ε*/(L·mol^−1^·cm^−1^)]: 248 (2.29) nm; IR (KBr) ν_max_: 3453 cm^−1^, 1620 cm^−1^; HR-ESI-MS *m*/*z* 337.1604 [M + Na]^+^ (calcd for C_16_H_26_O_6_Na, 337.1622). ^1^H and ^13^C NMR data are in Table 2.

### 3.4. Biological Assays

#### 3.4.1. Antibacterial Activity

The antibacterial activities of all compounds against 4 pathogenic bacteria (*Escherichia coli* (ATCC 25922), *Staphylococcus aureus* (ATCC 29213), *Enterococcus faecalis* (ATCC 29212), and *Stenotrophomonas maltophilia* (ATCC 17808)) were determined by the microplate assay method [27]. The maximum tested concentration was undertaken at 64 μg/mL. The MIC value expressed the lowest concentration of sample that inhibited bacterial growth. The broth medium containing pathogenic bacteria was used as the blank group and DMSO as the negative control. Vancomycin and meropenem, which were used as positive controls for bacteria, respectively, displayed the MIC values of 1.25 μg/mL against both *S. aureus* and *E. faecalis* and MIC values of 0.03 μg/mL against *E. coli* and *S. maltophilia*.

#### 3.4.2. Cytotoxic Activity

The cytotoxic activity of compounds **1**–**5** against MCF-7 (human breast cancer cell line), A549 (human non small cell lung cancer cell), and Hela (human cervical cancer cell) was determined using MTT assays [28]. The maximum tested concentration was undertaken at 100 μM. In a 96-well plate, each well was plated with 0.5–1 × 10^4^ cells (depending on the cell multiplication rate). The test compounds showed good solubilities in DMSO and did not precipitate when added to the cells. Density value (OD) was processed with the data at a wavelength of 570 nm, and the inhibition rate was calculated according to the formula: inhibition rate (%) = [1 − (OD sample/OD control)] × 100%. The positive controls were doxorubicin for MCF-7, A549, and Hela cells (the respective inhibition rates were 93.25%, 95.42%, and 92.16%).

#### 3.4.3. NO Inhibitory Activity

The cytotoxicity against RAW264.7 cells was evaluated by MTT method [26]. Concisely, 96-well plates containing cells at a density of 8 × 10^4^ cells/well were incubated at 37 °C. NO content of the supernatant was checked by Griess reagent, similar to the previous publication [29]. In brief, RAW264.7 cells in a 96-well plate (2 × 10^5^ cells/well) were incubated overnight. Cells were treated with different concentrations of samples (1 h) first, then incubated with LPS (2 µg/mL) for 24 h. After incubation, the cell supernatant (50 µL) was transferred to another 96-well plate and commingled with Griess reagent (*v*/*v*, 1:1) for 10 min incubation at an indoor temperature. The absorbance was measured at 570 nm using a microplate reader. NO production in the supernatant was assessed by Griess reagent. Dexamethasone served as a positive control. NO levels were tested by nitrite using the Griess reaction. The nitrite concentration was calculated from the standard curve. We diluted 1 mM NaNO_2_ in standard solution to gain the final concentrations of 1, 2, 5, 10, 20, 40, 60, and 100 µM and added 50.0 μL to a 96-well plate. Then, 50.0 μL of Griess reagent was added for 10 min at 37 °C. Absorbance was measured at 540 nm. We then used DRAW to create a standard curve with concentration and absorbance as abscissa and ordinate, respectively.

## 4. Conclusions

In this study, four previously undescribed pyranone derivatives, named ascomycopyrones A–D (**1**–**4**), as well as one known analogue simplicilopyrone (**5**), were isolated from the endophytic fungus *Ascomycota* sp. FAE17 derived from the flowers of *S. formosa*. The structures of these pyranones with absolute configurations were determined unambiguously by NMR spectroscopic data analysis and quantum chemical calculations. Compounds **1**–**5** were tested for antibacterial, cytotoxic activity, and NO inhibitory activity. The results showed that all isolates exhibited weak antibacterial activity (MIC values all over 64 μg/mL) against *E*. *coli*, *S. aureus*, *E. faecalis*, and *S. maltophilia*, without significant activity against MCF-7, A549, and Hela cells at the maximum tested concentration of 100 µM and showed no significant inhibitory activity against NO production at the concentration of 50 µM.

## Figures and Tables

**Table 1:** ^1^H (400 MHz) and ^13^C NMR (100 MHz) Data for **1**–**2** (DMSO-*d*_6_).

Position	1		2	
	*δ*_C_	*δ*_H_ (*J* in Hz)	*δ*_C_	*δ*_H_ (*J* in Hz)
2	165.8		165.7	
3	91.0	5.22, s	91.1	5.22, s
4	174.2		173.2	
5	38.7	2.49, m	38.7	2.49, m
6	67.1	4.32, d (3.6)	67.1	4.32, d (3.2)
7	56.2	3.73, s	56.2	3.70, s
8	33.3	2.17, m	33.3	2.17, m
9	66.4	3.96, dd (11.2, 6.4); 3.91, dd (11.2, 6.4)	67.0	4.00, dd (11.2, 6.0); 3.92, dd (11.2, 6.0)
10	14.3	0.93, d (7.2)	14.3	0.93, d (7.2)
1′	170.3			
2′	20.6	2.00, s	165.9	
3′			91.2	5.22, s
4′			173.4	
5′			38.9	2.86, m
6′			67.2	4.38, m; 4.19, dd (11.2, 3.2)
7′			56.4	3.72, s
8′			39.2	2.83, m
9′			174.3	
10′			15.7	1.08, d (6.8)

s-single; m-multiplet; d-doublet; dd-double doublet.

**Table 2:** ^1^H (400 MHz) and ^13^C NMR (100 MHz) data for **3**–**4** (DMSO-*d*_6_).

Position	3		4	
	*δ*_C_	*δ*_H_ (*J* in Hz)	*δ*_C_	*δ*_H_ (*J* in Hz)
2	166.0		165.9	
3	90.7	5.18, s	90.6	5.18, s
4	174.9		175.0	
5	39.0	2.44, m	39.0	2.44, m
6	67.3	4.30, d (3.6)	67.5	4.31, d (3.6)
7	56.1	3.72, s	56.0	3.72, s
8	34.2	2.05, m	34.1	2.05, m
9	69.8	3.44, dd (9.6, 6.8); 3.25, dd (9.6, 6.8)	73.6	3.27, m
10	14.8	0.89, d (7.2)	14.8	0.91, d (6.8)
1′				
2′	110.8	4.58, d (2.4)	104.2	
3′	42.0	1.80, dt (6.8, 2.4)	54.8	1.57, ddd (10.2, 7.6, 6.0)
4′	53.9	1.59, m	35.8	2.08, m
5′	69.2	3.90, t (8.0); 3.66, t (8.8)	71.9	3.91, t (8.0) 3.19, m
6′	18.0	0.97, d (7.2)	26.6	1.34, s
7′	67.4	3.54, t (6.8)	70.4	3.52, m; 3.33, d (3.2)
8′	22.6	1.07, d (6.4)	17.5	0.99, d (6.8)

s-single; m -multiplet; t-triplet; d-doublet; dd-doublet of doublets; ddd-doublet of doublet of doublets; dt-doublet of triplets.

**Table 3:** Results of antibacterial activities.

MIC (μg/mL)				
Compounds	*S. aureus*	*E. faecalis*	*E. coli*	*S. maltophilia*
**1**	>64	>64	>64	>64
**2**	>64	>64	>64	>64
**3**	>64	>64	>64	>64
**4**	>64	>64	>64	>64
**5**	>64	>64	>64	>64
vancomycin	1.25	1.25		
meropenem			0.03	0.03

**Table 4:** Results of antitumor activities.

Inhibition Rate (%)			
Compounds	MCF-7	A549	Hela
**1**	13.21	21.04	8.13
**2**	8.19	10.23	13.11
**3**	16.28	31.22	24.56
**4**	5.36	2.79	6.35
**5**	20.13	12.65	17.03
adriamycin	93.25	95.42	92.16

## Data Availability

The data presented in this study are available in Supplementary Materials.

## Supplementary Materials

The following supporting information can be downloaded at: https://www.mdpi.com/article/10.3390/molecules28145388/s1, Figures S1–S32: 1D, 2D NMR spectra and HR-ESI-MS of compounds **1**–**4**.

## Sample Availability

Samples of the compounds are not available from the authors.
