# Supplementary material for: Four Undescribed Pyranones from the Scutellaria formosana-Derived Endophytic Fungi Ascomycota sp. FAE17
Source: Molecules. 2023 Jul 13;28(14):5388. doi: 10.3390/molecules28145388 (PMC10383492; doi:10.3390/molecules28145388)
Supplement: Supplementary file 1 [file molecules-28-05388-s001.zip › molecules-2452927-supplementary.pdf]

SUPPLEMENTARY MATERIALS

Four Undescribed Pyranones from the *Scutellaria formosana*-Derived Endophytic Fungi *Ascomycota* sp. FAE17

Jianni Yang [1,2,†], Yang Hui [1,2,†], Zhaoxia Chen [1,2], Guangying Chen [1,2], Xiaoping Song [1,2], Zhenfan Sun [3], Changri Han [3,*] and Wenhao Chen [1,2,*]

[1] Key Laboratory of Tropical Medicinal Resource Chemistry of Ministry of Education, College of Chemistry and Chemical Engineering, Hainan Normal University, Haikou 571158, China; jianni1216@163.com (J.Y.); 070066@hainnu.edu.cn (Y.H.); 17689849894@163.com (Z.C.); chgying123@163.com (G.C.); sxp628@126.com (X.S.)

[2] Key Laboratory of Tropical Medicinal Plant Chemistry of Hainan Province, Haikou 571158, China

[3] Key Laboratory of Medicinal and Edible Plants Resources of Hainan Province, Hainan Vocational University of Science and Technology, Haikou 571158, China; sunzhenf@163.com

* Correspondence: hchr116@hvust.edu.cn (C.H.); 070103@hainnu.edu.cn (W.C.)

† These authors contributed equally to this work.

Contents

Figure S1. ^{1}H NMR spectrum of **1** in DMSO-d_6 3

Figure S2. ^{13}C NMR spectrum of **1** in DMSO-d_6 3

Figure S3. 135-DEPT spectrum of **1** in DMSO-d_6 4

Figure S4. HSQC spectrum of **1** in DMSO-d_6 4

Figure S5. ^{1}H-^{1}H COSY spectrum of **1** in DMSO-d_6 5

Figure S6. HMBC spectrum of **1** in DMSO-d_6 5

Figure S7. NOESY spectrum of **1** in DMSO-d_6 6

Figure S8. HRESIMS spectrum of **1** 6

Figure S9. ^{1}H NMR spectrum of **2** in DMSO-d_6 7

Figure S10. ^{13}C NMR spectrum of **2** in DMSO-d_6 7

Figure S11. 135-DEPT spectrum of **2** in DMSO-d_6 8

Figure S12. HSQC spectrum of **2** in DMSO-d_6 8

Figure S13. ^{1}H-^{1}H COSY spectrum of **2** in DMSO-d_6 9

Figure S14. HMBC spectrum of **2** in DMSO-d_6 9

Figure S15. NOESY spectrum of **2** in DMSO-d_6 10

Figure S16. HRESIMS spectrum of **2** 10

Figure S17. ^{1}H NMR spectrum of **3** in DMSO-d_6 11

Figure S18. ^{13}C NMR spectrum of **3** in DMSO-d_6 11

Figure S19. 135-DEPT spectrum of **3** in DMSO-d_6 12

Figure S20. HSQC spectrum of **3** in DMSO-d_6 12

Figure S21. ^{1}H-^{1}H COSY spectrum of **3** in DMSO-d_6 13

Figure S22. HMBC spectrum of **3** in DMSO-d_6 13
Figure S23. NOESY spectrum of **3** in DMSO-d_6 14
Figure S24. HRESIMS spectrum of **3** 14
Figure S25. ^{1}H NMR spectrum of **4** in DMSO-d_6 15
Figure S26. ^{13}C NMR spectrum of **4** in DMSO-d_6 15
Figure S27. 135-DEPT spectrum of **4** in DMSO-d_6 16
Figure S28. HSQC spectrum of **4** in DMSO-d_6 16
Figure S29. ^{1}H-^{1}H COSY spectrum of **4** in DMSO-d_6 17
Figure S30. HMBC spectrum of **4** in DMSO-d_6 17
Figure S31. NOESY spectrum of **4** in DMSO-d_6 18
Figure S32. HRESIMS spectrum of **4** 18

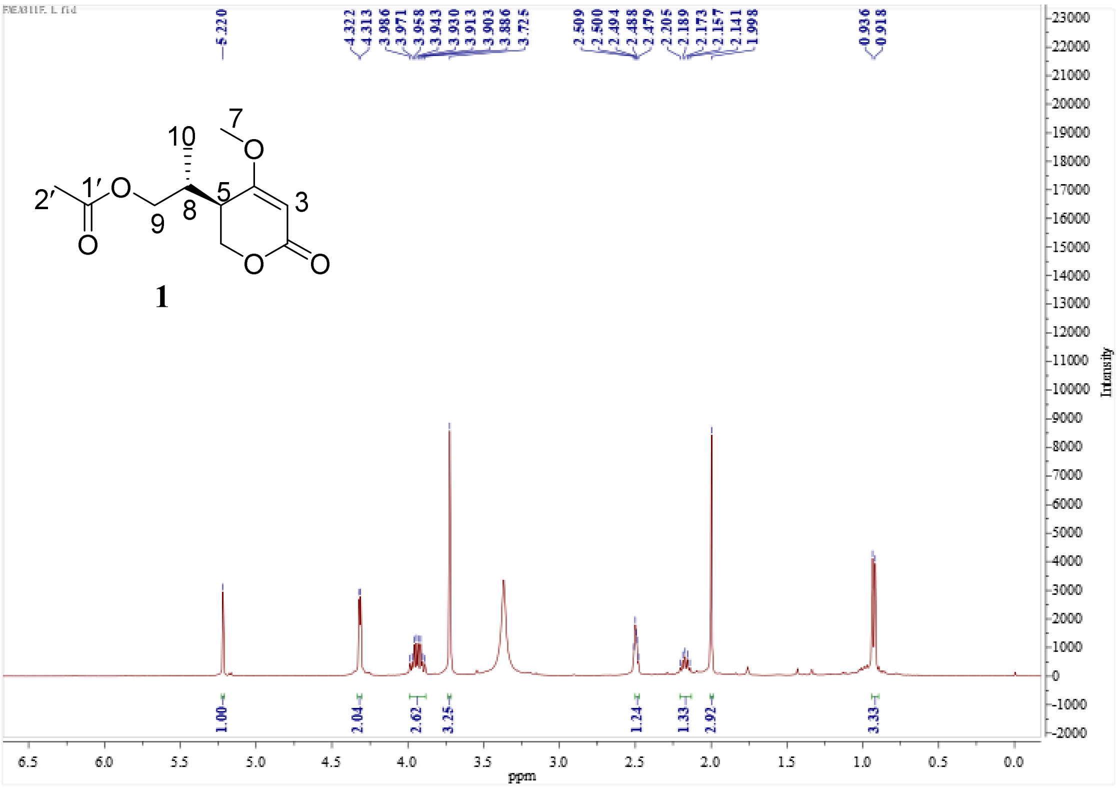

Figure S1. ^{1}H NMR spectrum of **1** in DMSO-d_6

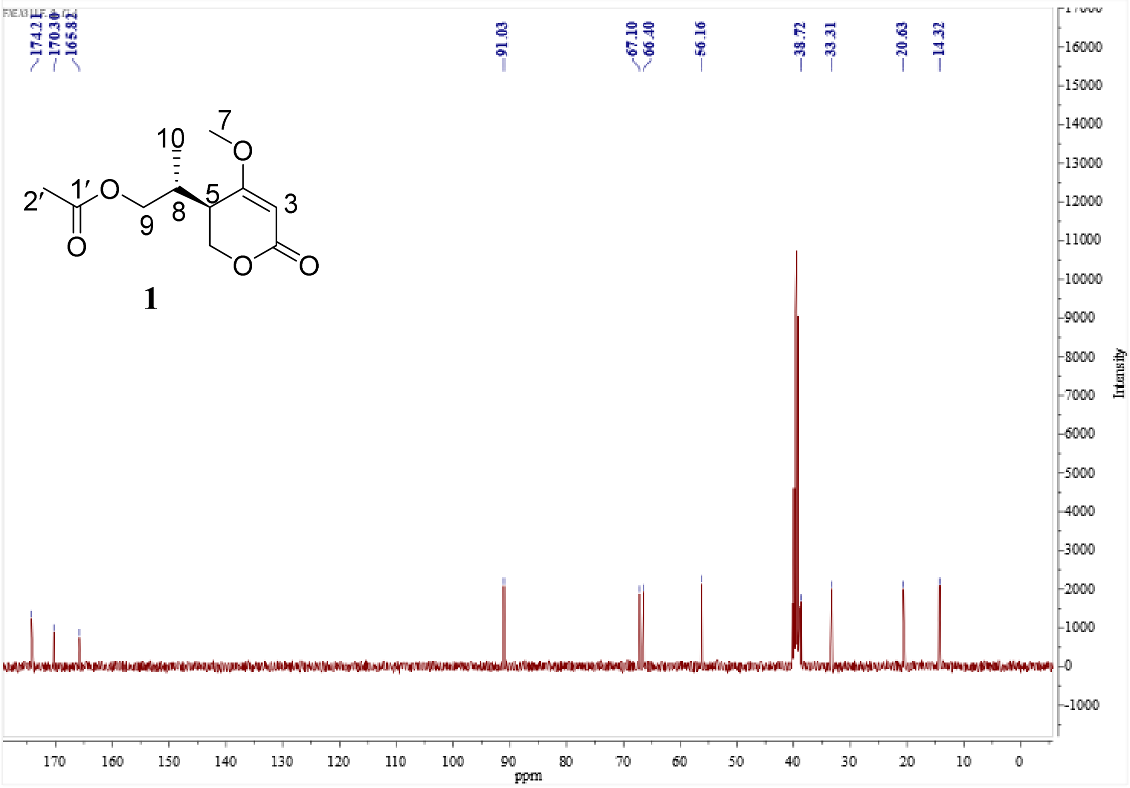

Figure S2. ^{13}C NMR spectrum of **1** in DMSO-d_6

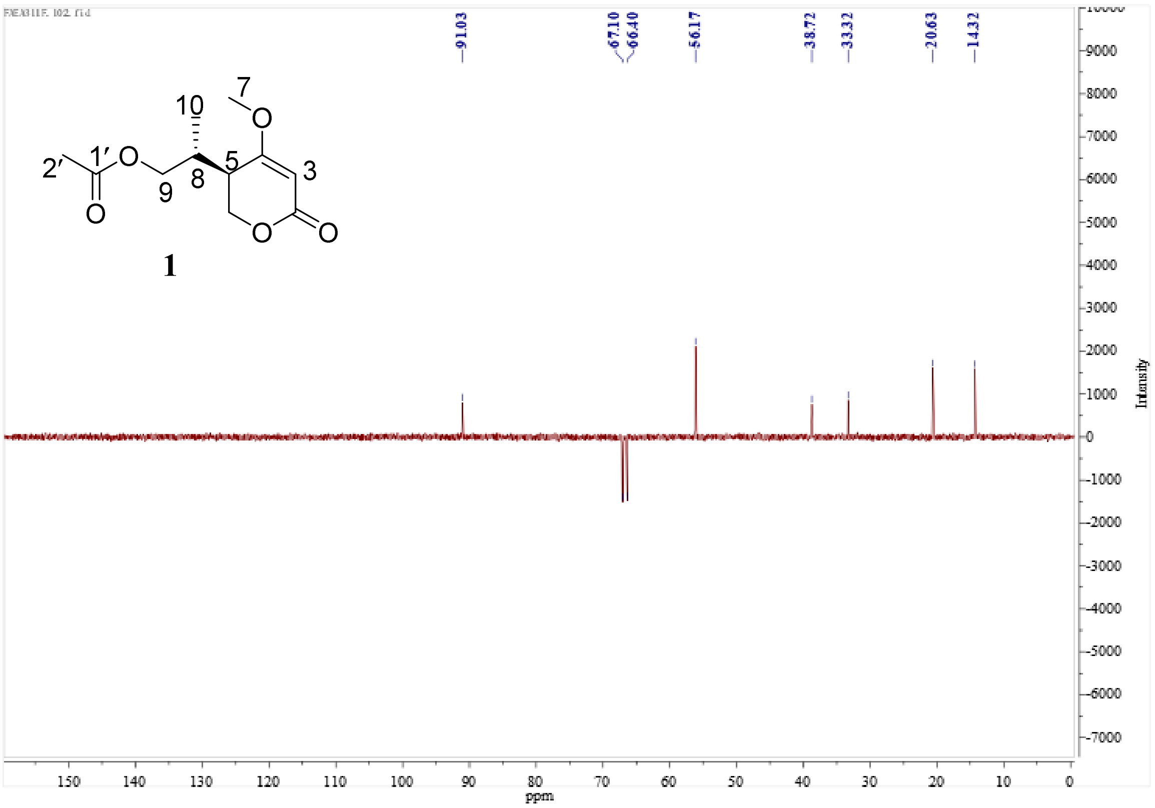

Figure S3. 135-DEPT spectrum of **1** in DMSO-d_6

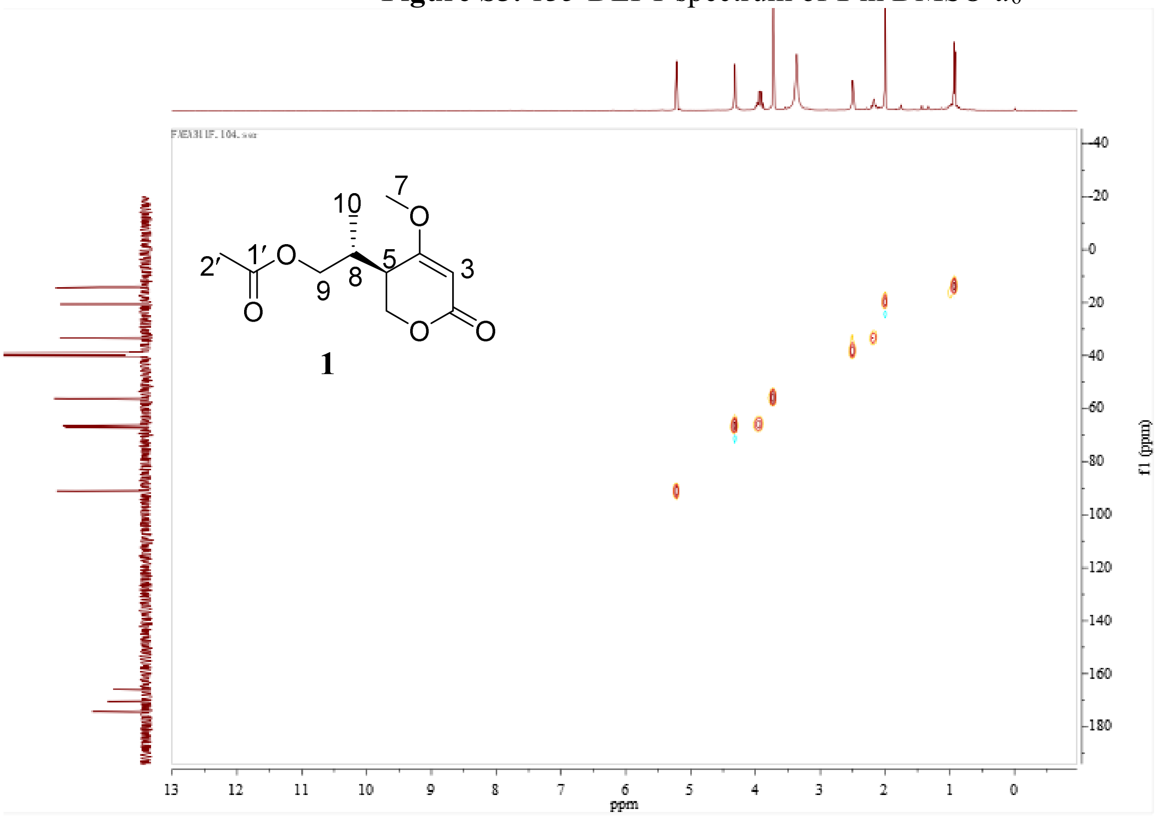

Figure S4. HSQC spectrum of **1** in DMSO-d_6

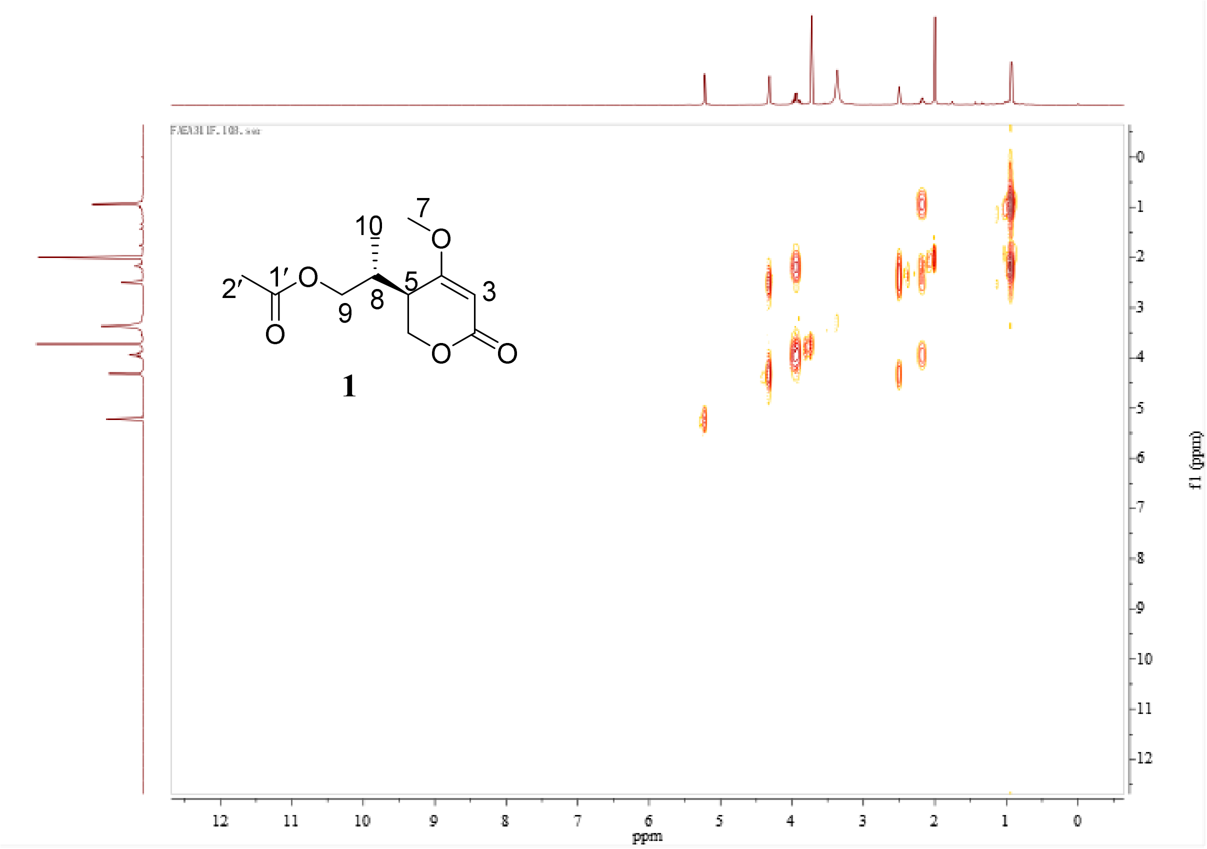

Figure S5. ^{1}H-^{1}H COSY spectrum of **1** in DMSO-d_6

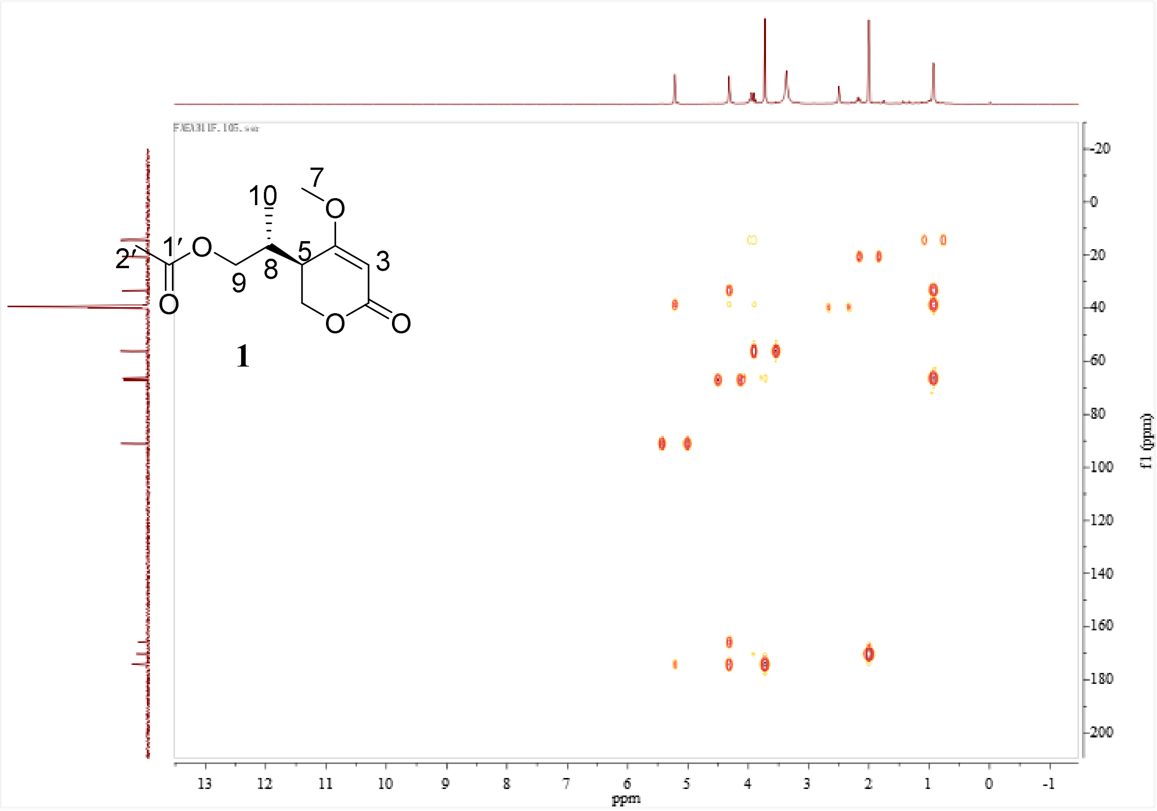

Figure S6. HMBC spectrum of **1** in DMSO-d_6

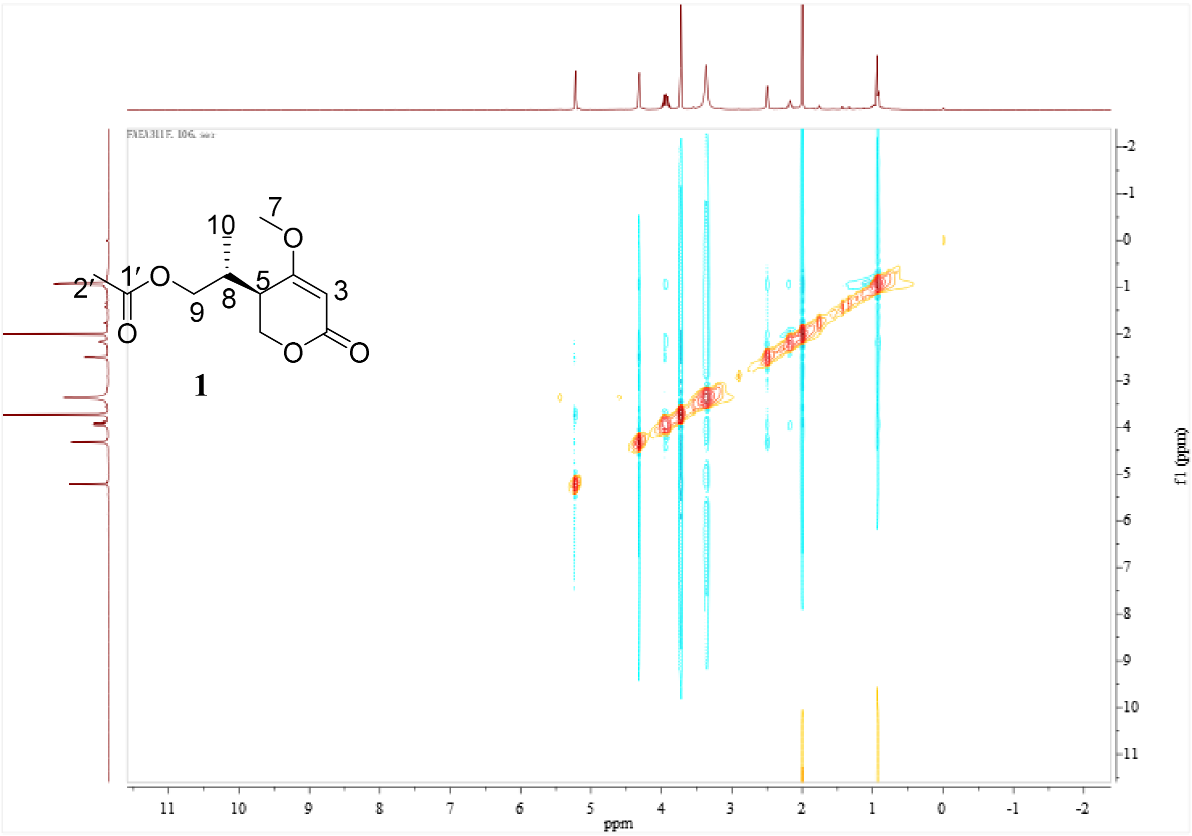

Figure S7. NOESY spectrum of **1** in DMSO-d_6

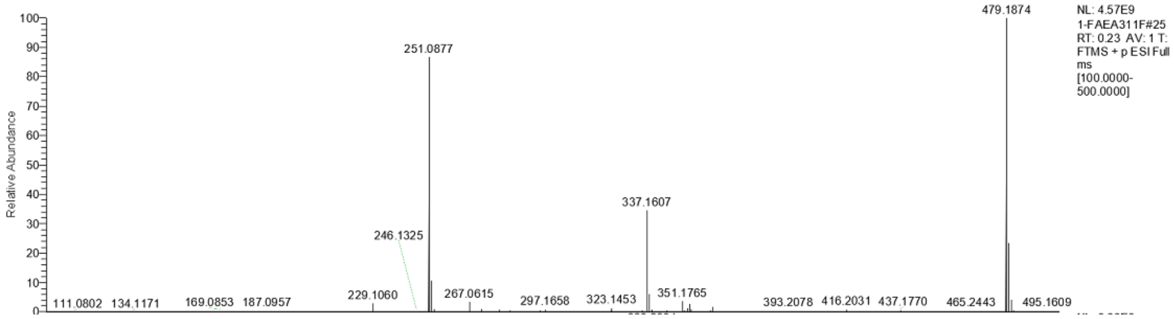

Figure S8. HRESIMS spectrum of **1**

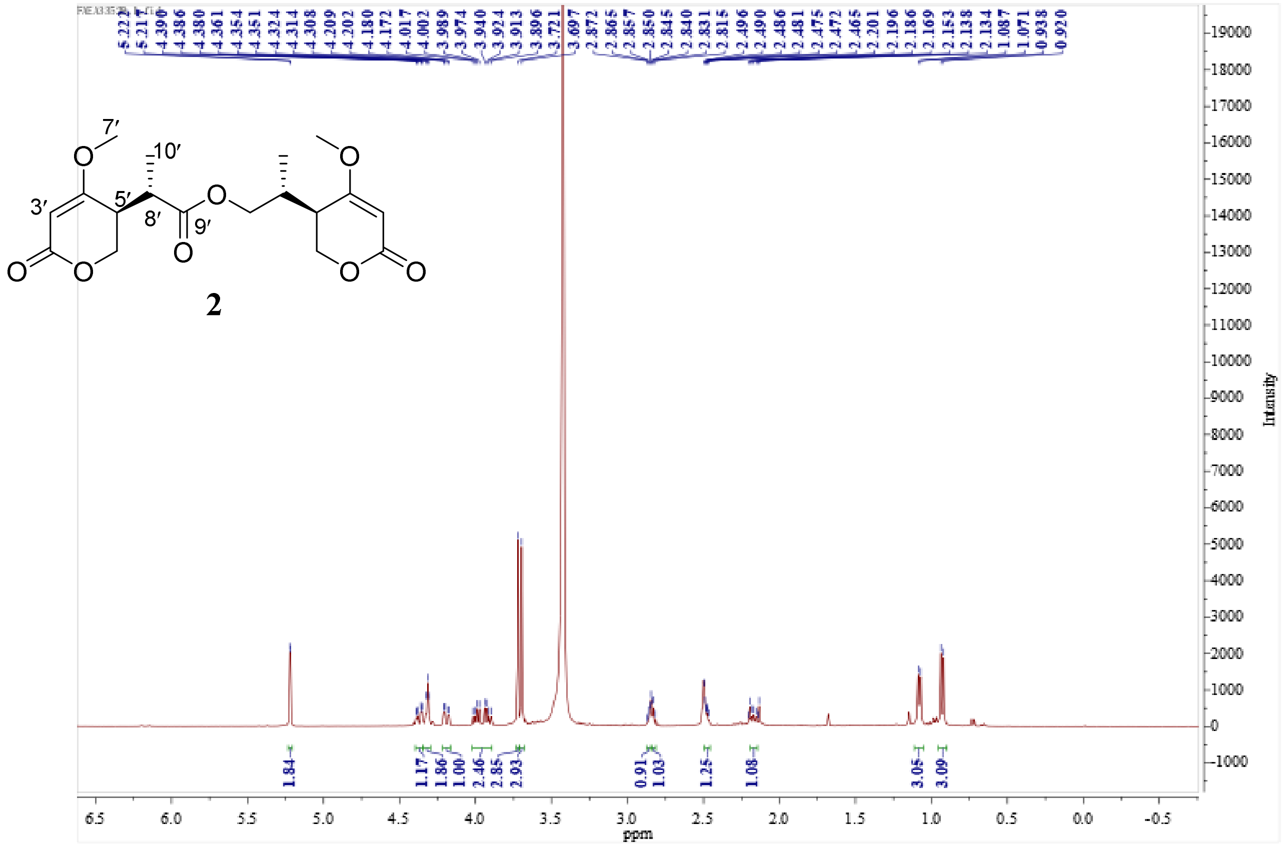

Figure S9. ^{1}H NMR spectrum of **2** in DMSO-d_6

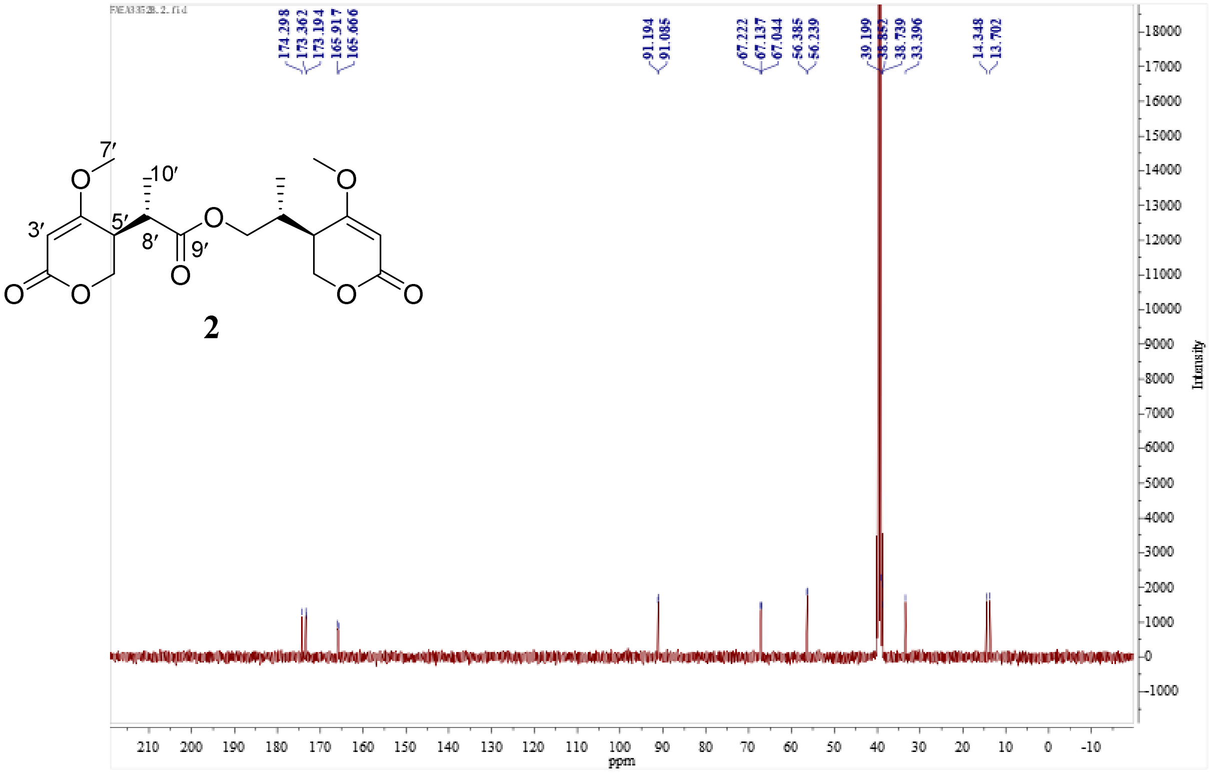

Figure S10. ^{13}C NMR spectrum of **2** in DMSO-d_6

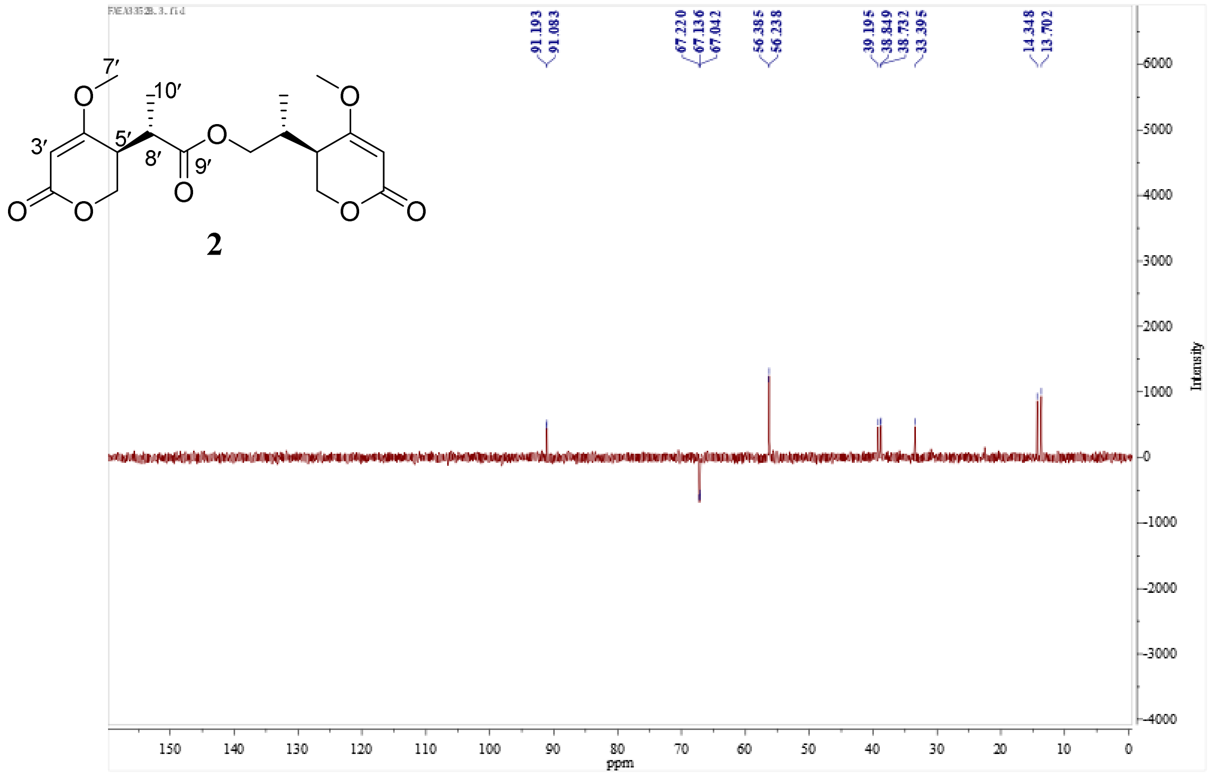

Figure S11. 135-DEPT spectrum of **2** in DMSO-d_6

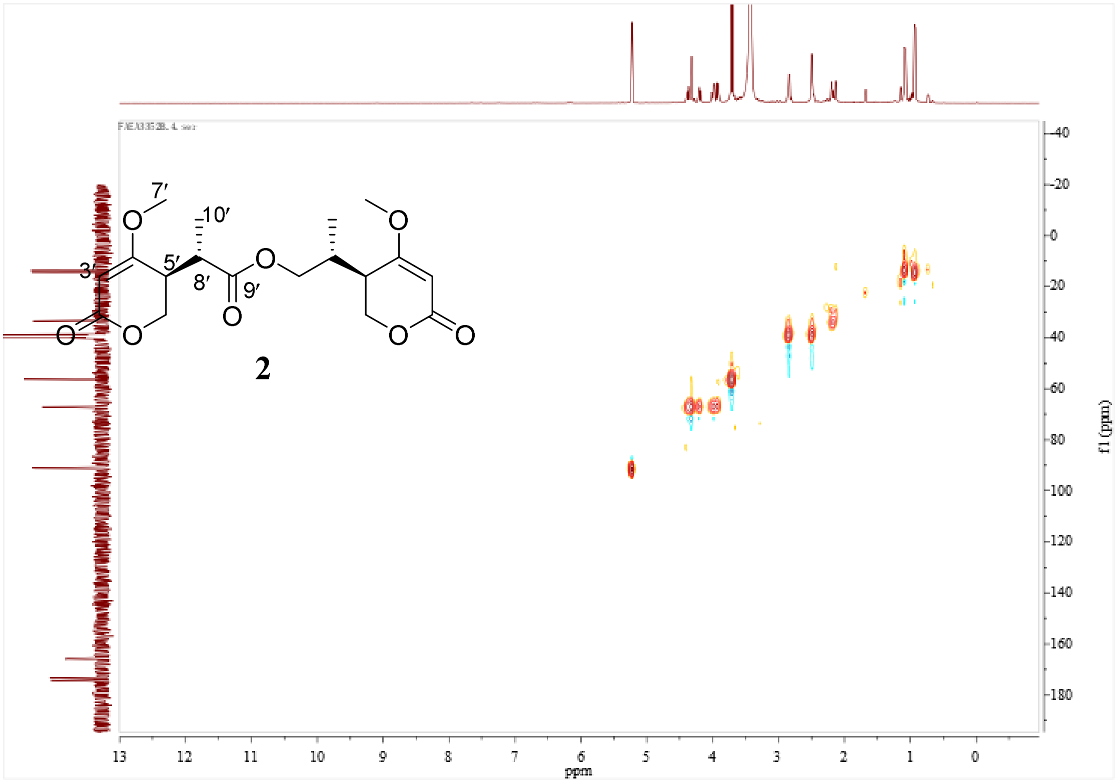

Figure S12. HSQC spectrum of **2** in DMSO-d_6

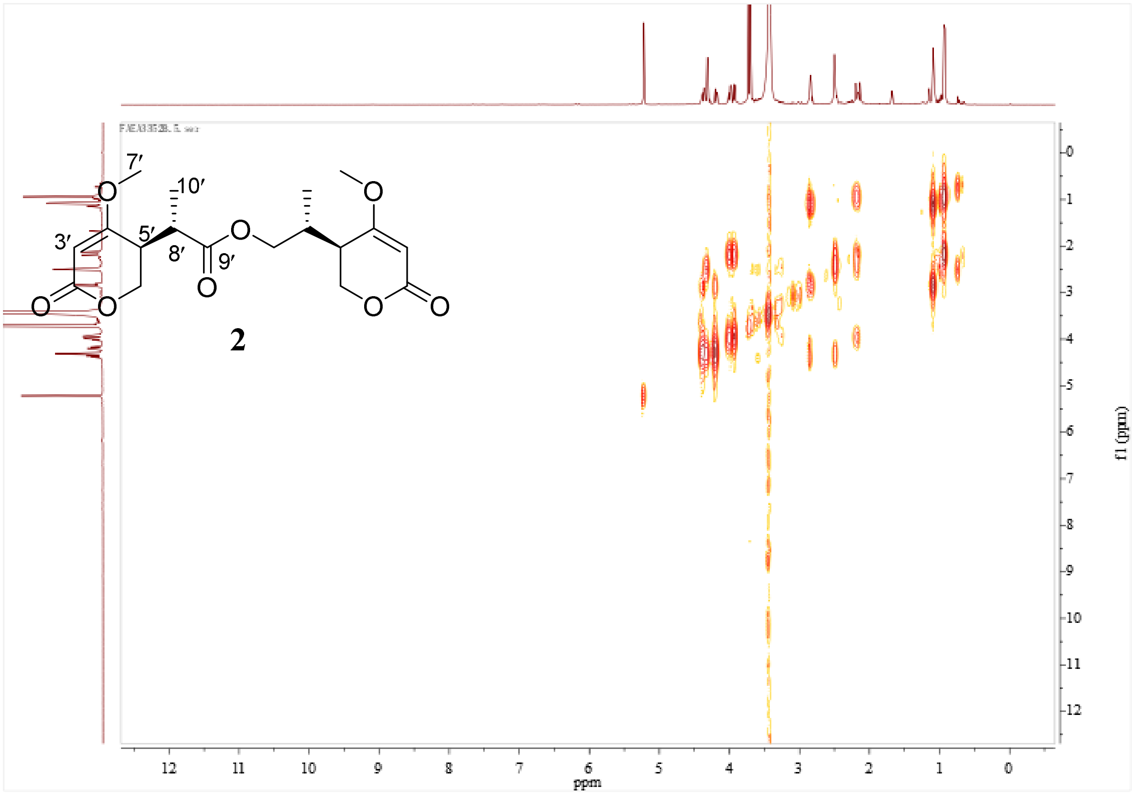

Figure S13. ^{1}H-^{1}H COSY spectrum of **2** in DMSO-d_6

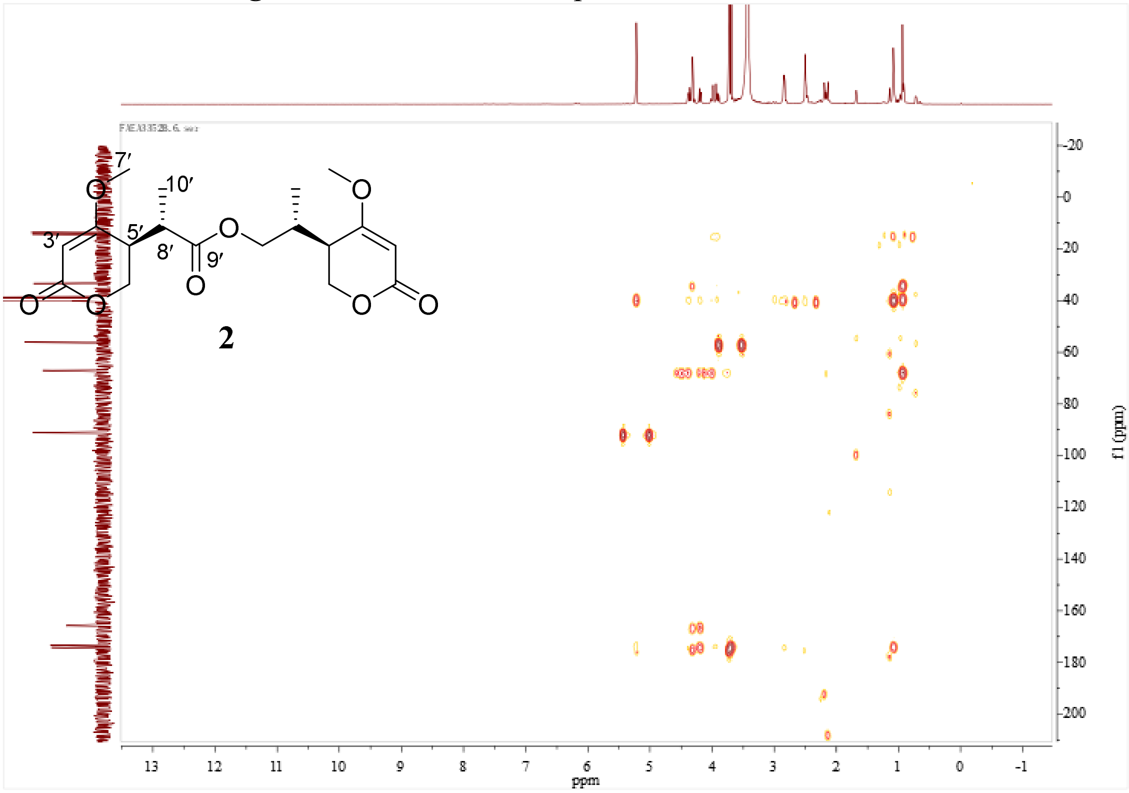

Figure S14. HMBC spectrum of **2** in DMSO-d_6

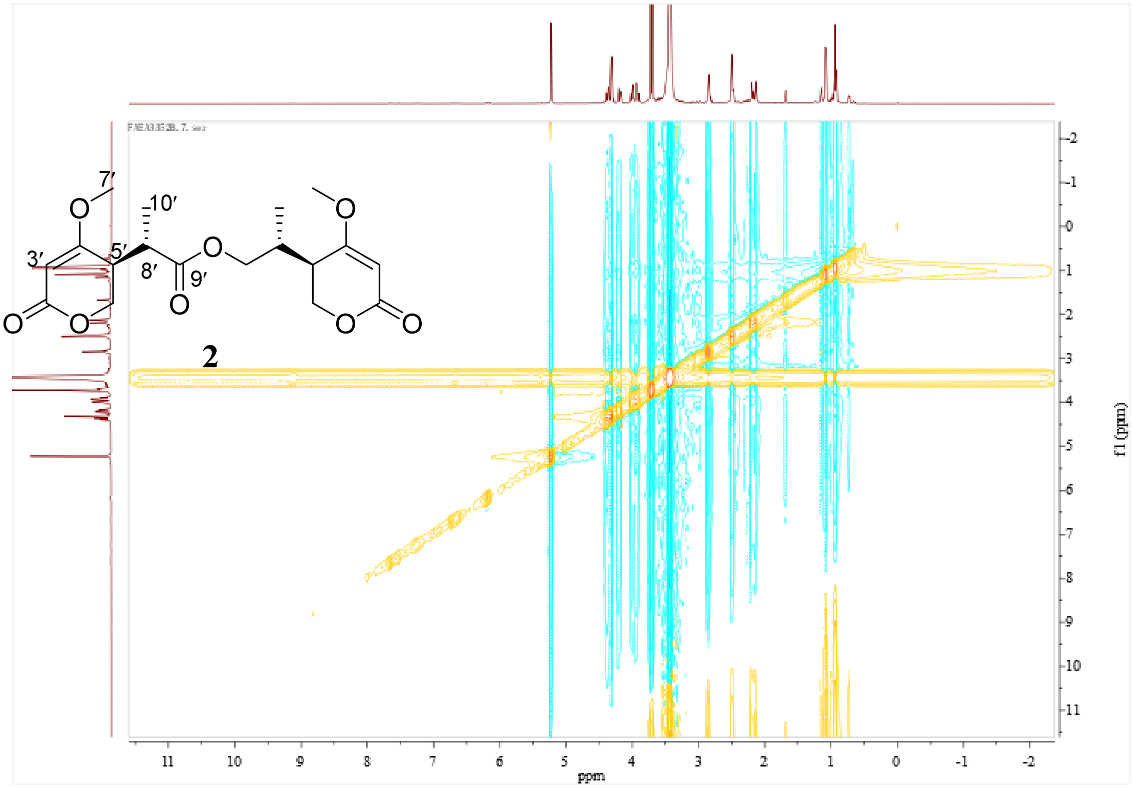

Figure S15. NOESY spectrum of **2** in DMSO-d_6

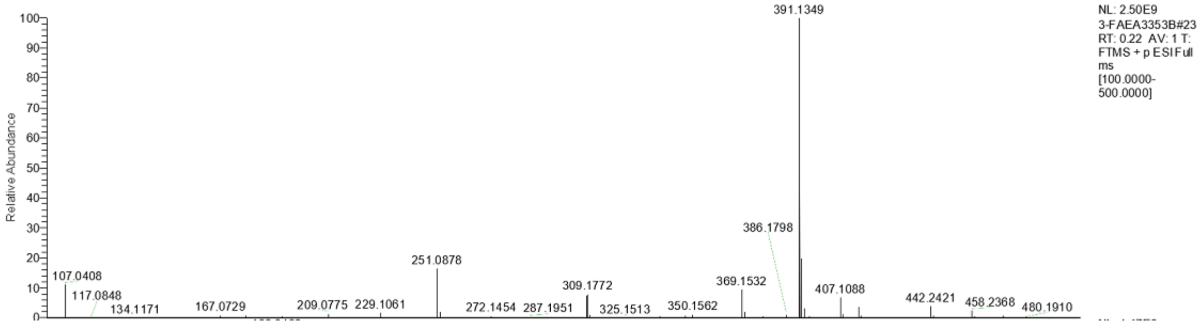

Figure S16. HRESIMS spectrum of **2**

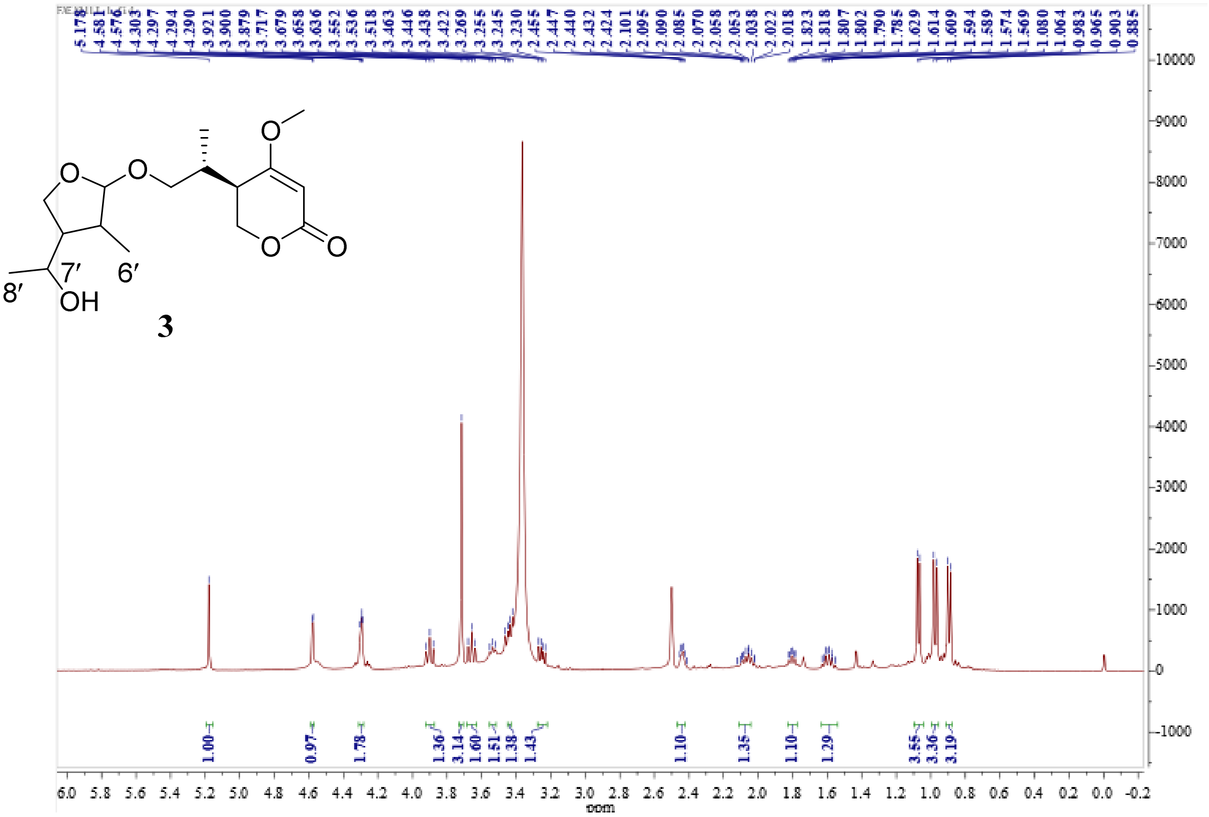

Figure S17. ^{1}H NMR spectrum of **3** in DMSO-d_6

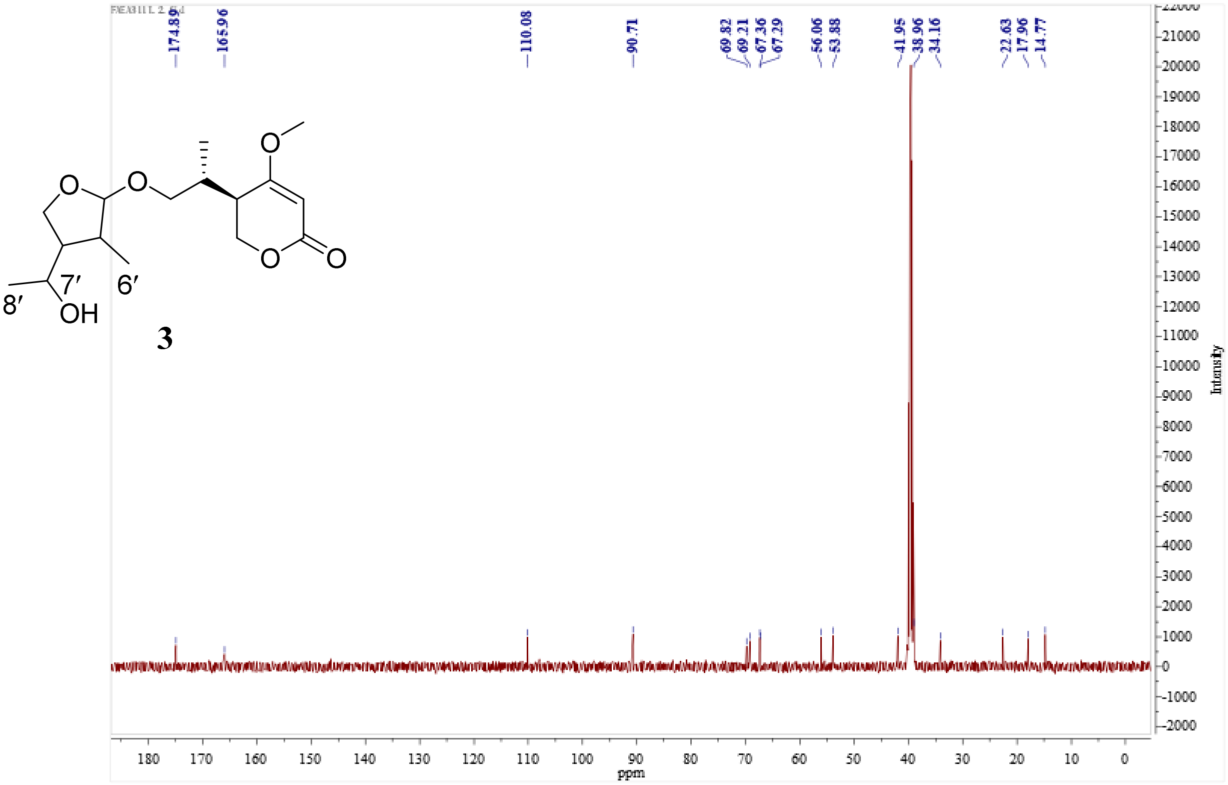

Figure S18. ^{13}C NMR spectrum of **3** in DMSO-d_6

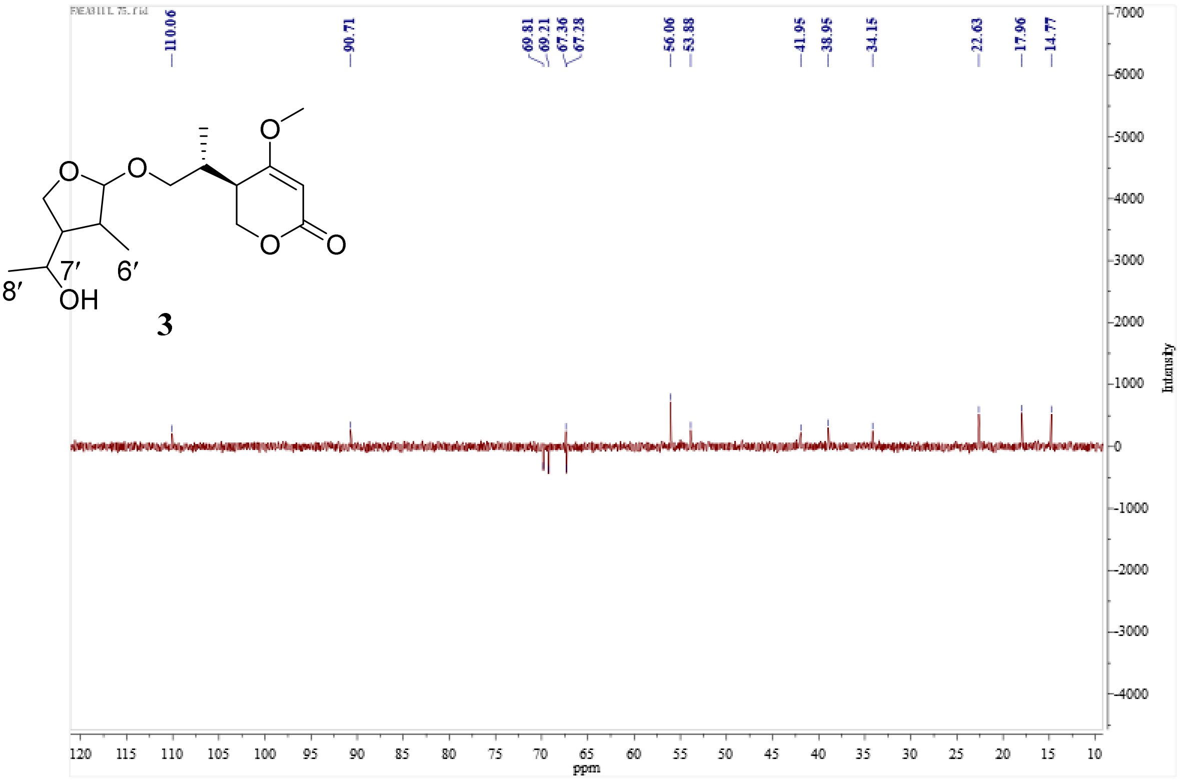

Figure S19. 135-DEPT spectrum of **3** in DMSO-d_6

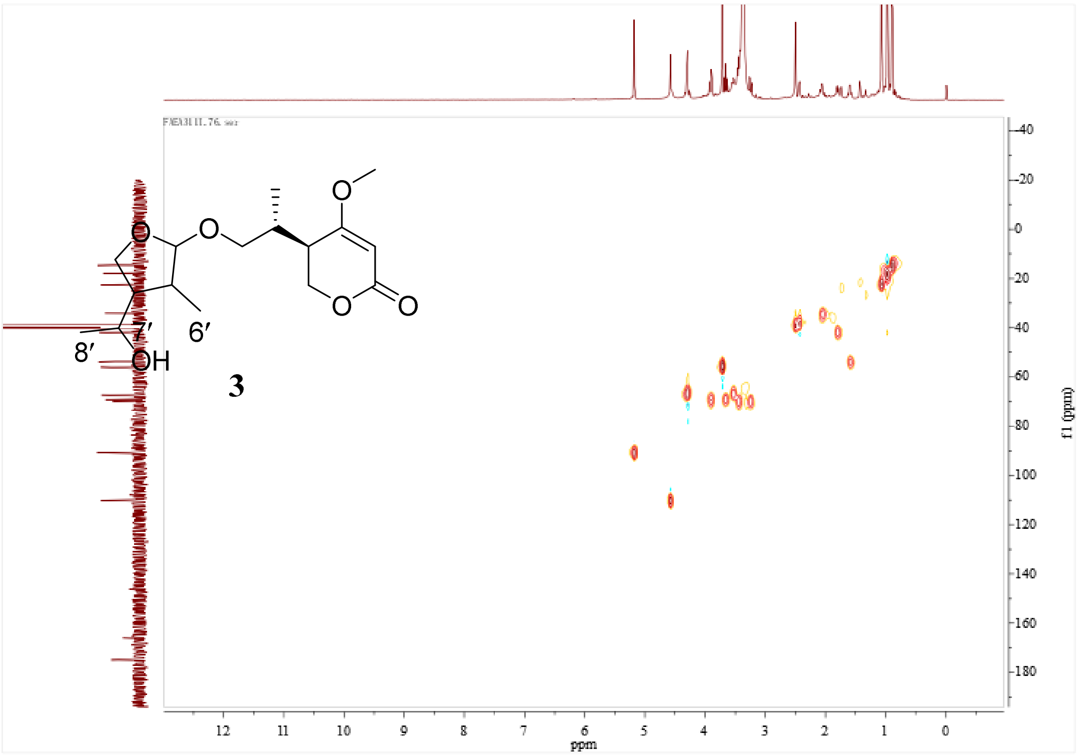

Figure S20. HSQC spectrum of **3** in DMSO-d_6

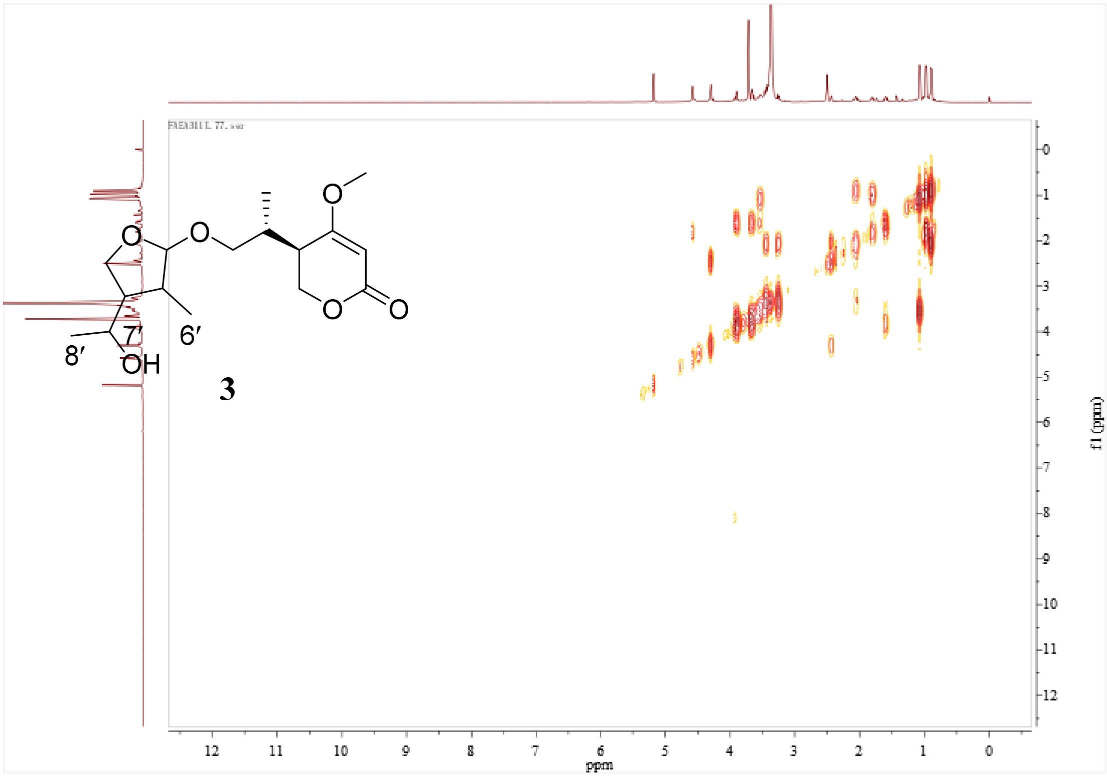

Figure S21. ^{1}H-^{1}H COSY spectrum of **3** in DMSO-d_6

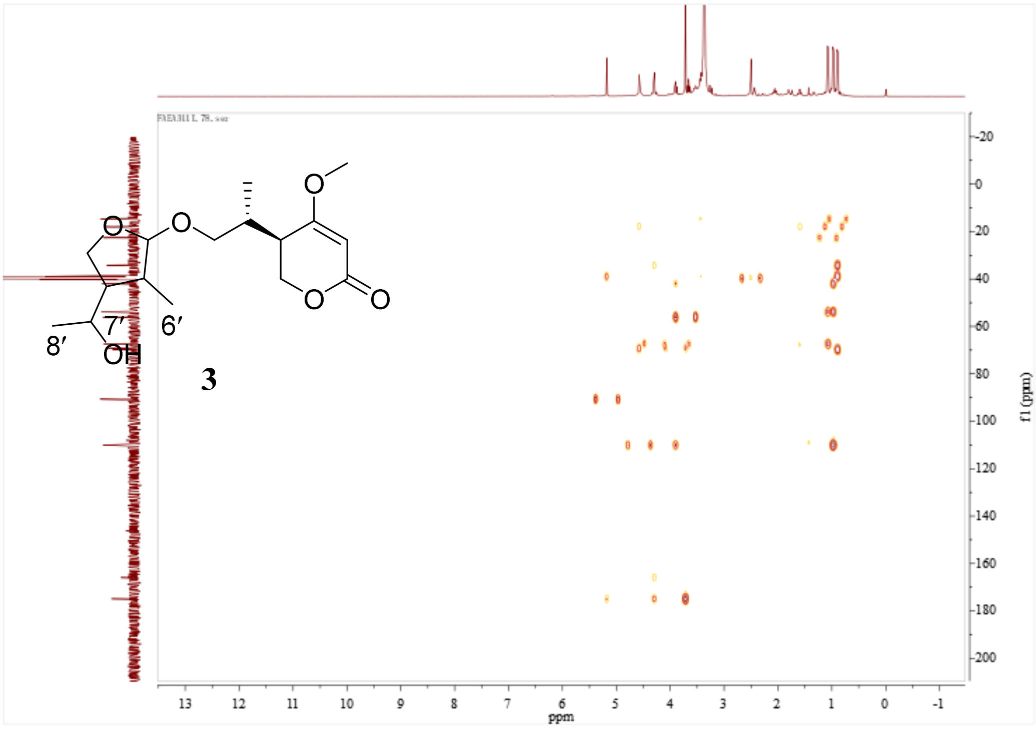

Figure S22. HMBC spectrum of **3** in DMSO-d_6

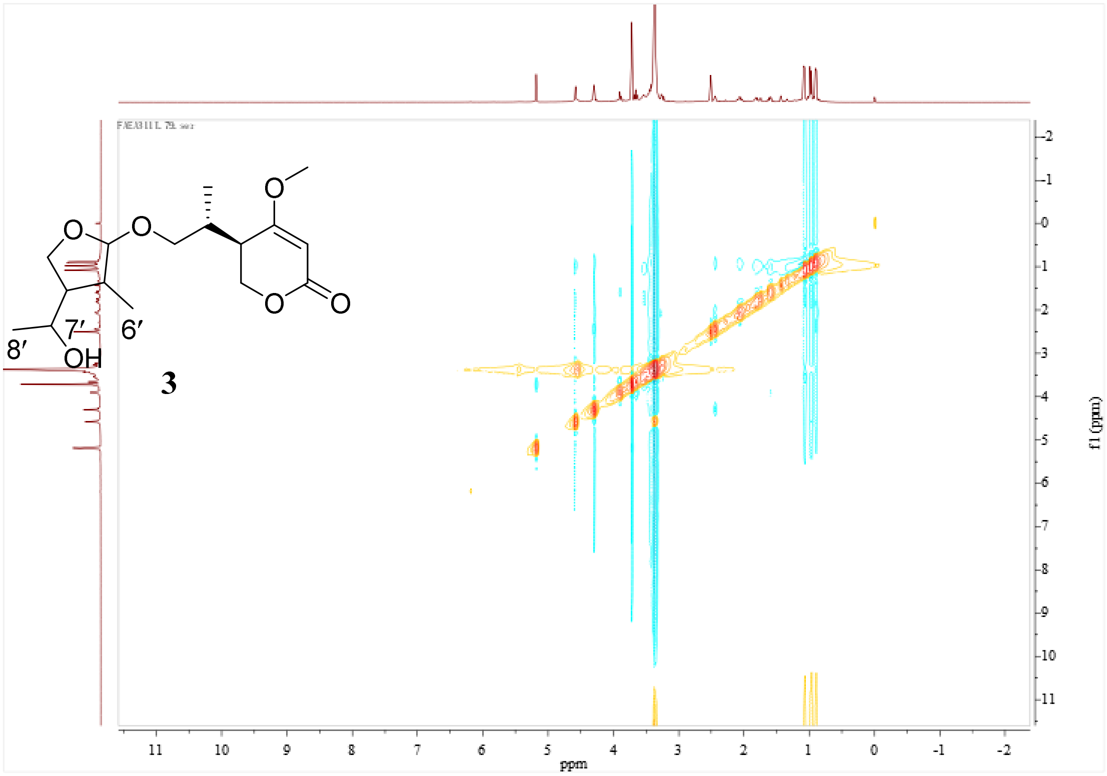

Figure S23. NOESY spectrum of **3** in DMSO-d_6

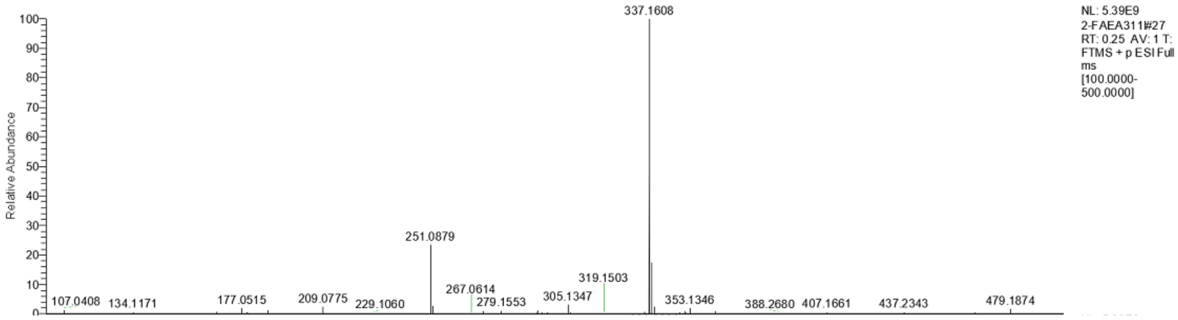

Figure S24. HRESIMS spectrum of **3**

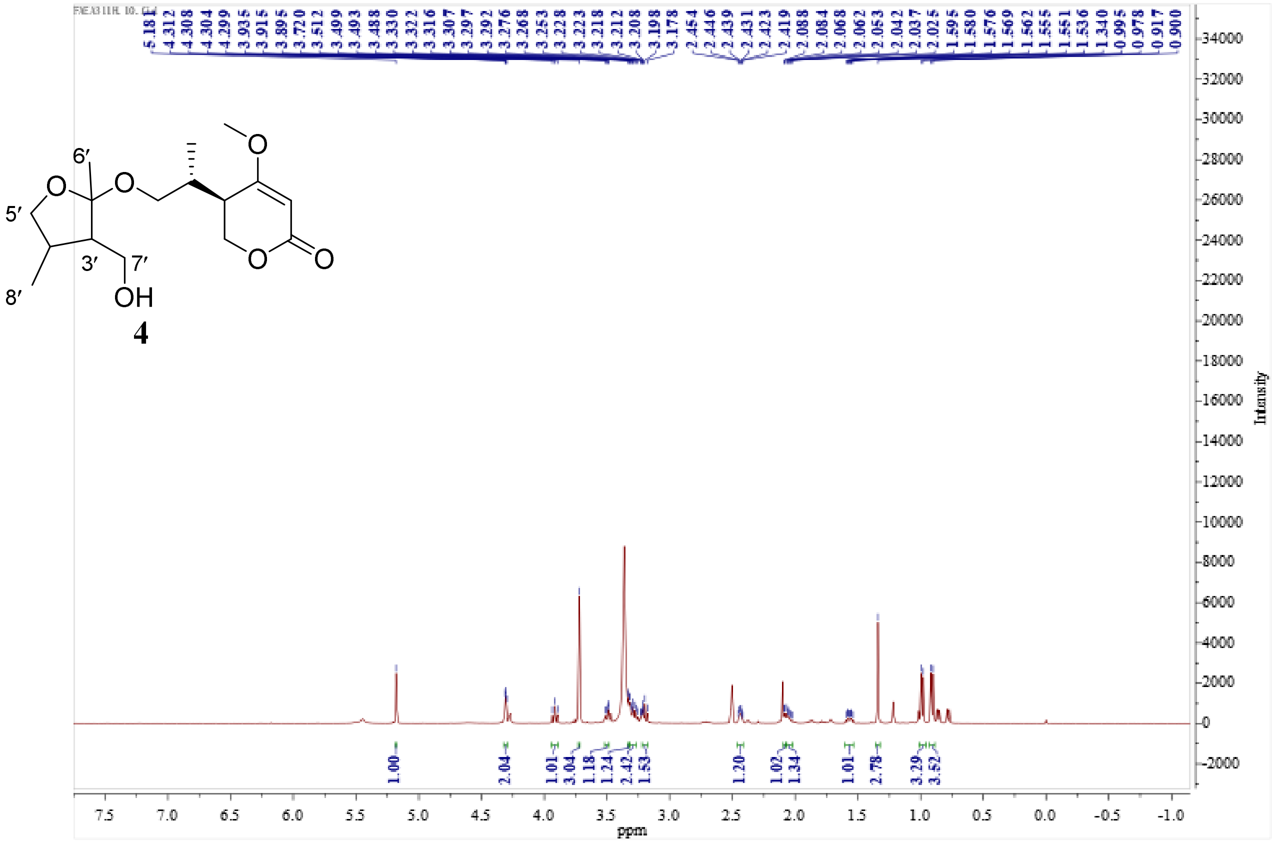

Figure S25. ^{1}H NMR spectrum of **4** in DMSO-d_6

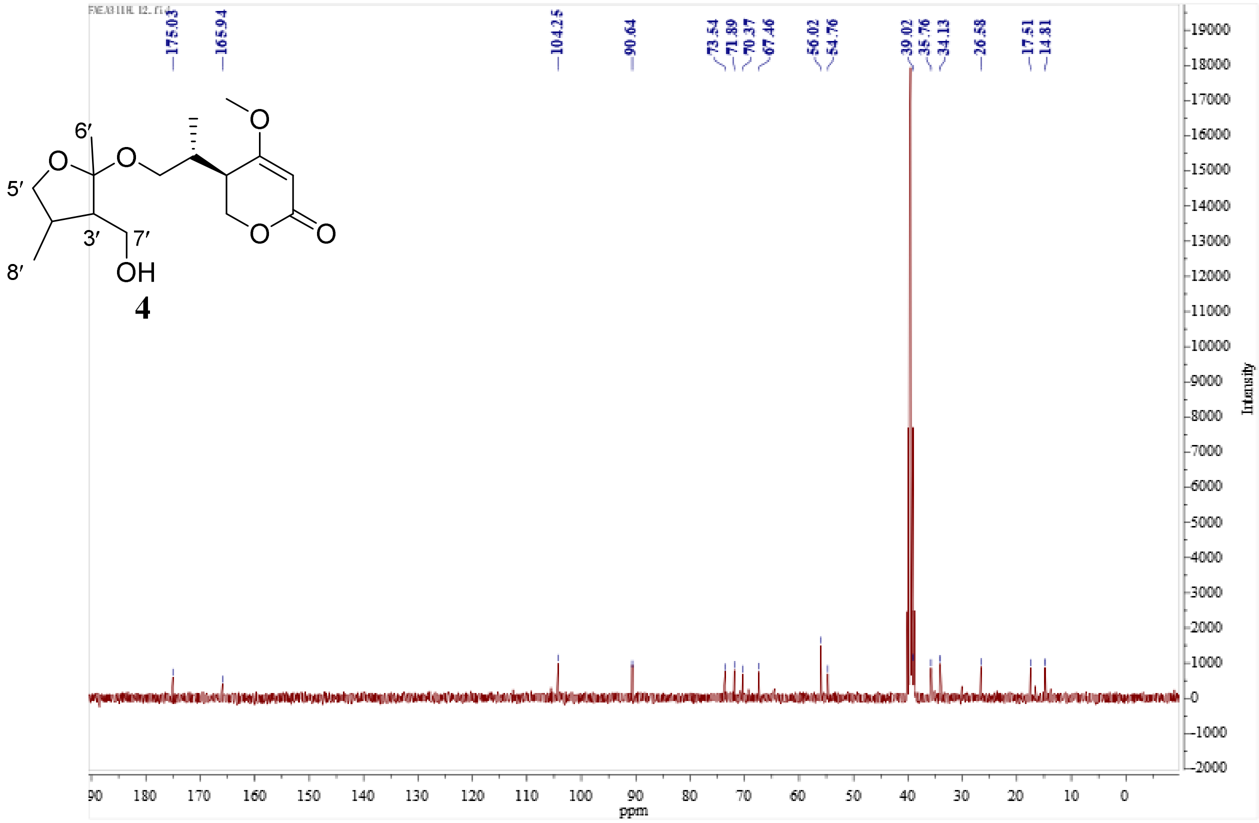

Figure S26. ^{13}C NMR spectrum of **4** in DMSO-d_6

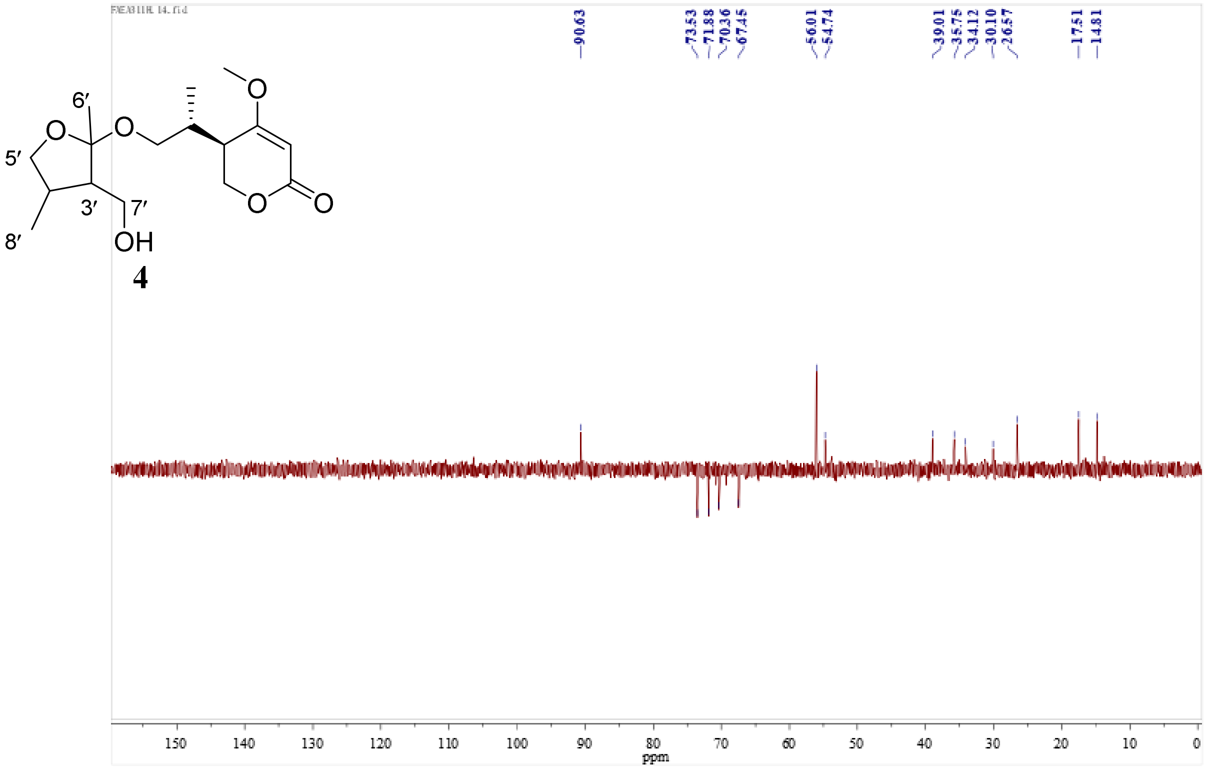

Figure S27. 135-DEPT spectrum of **4** in DMSO-d_6

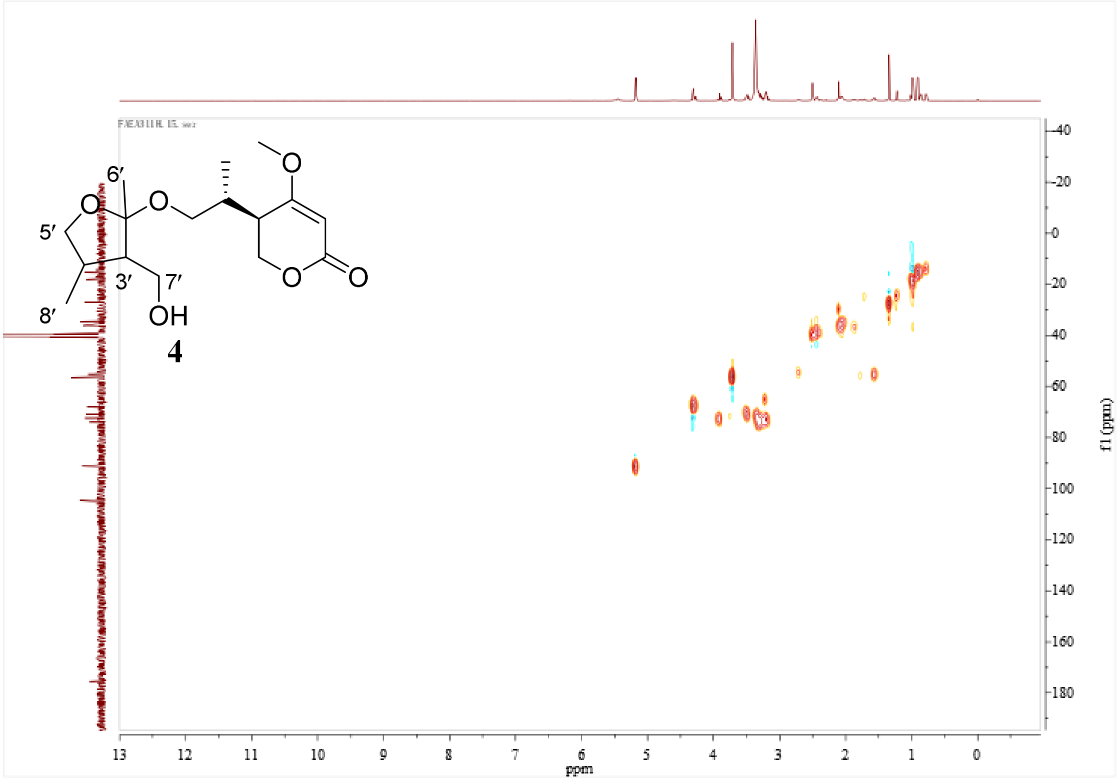

Figure S28. HSQC spectrum of **4** in DMSO-d_6

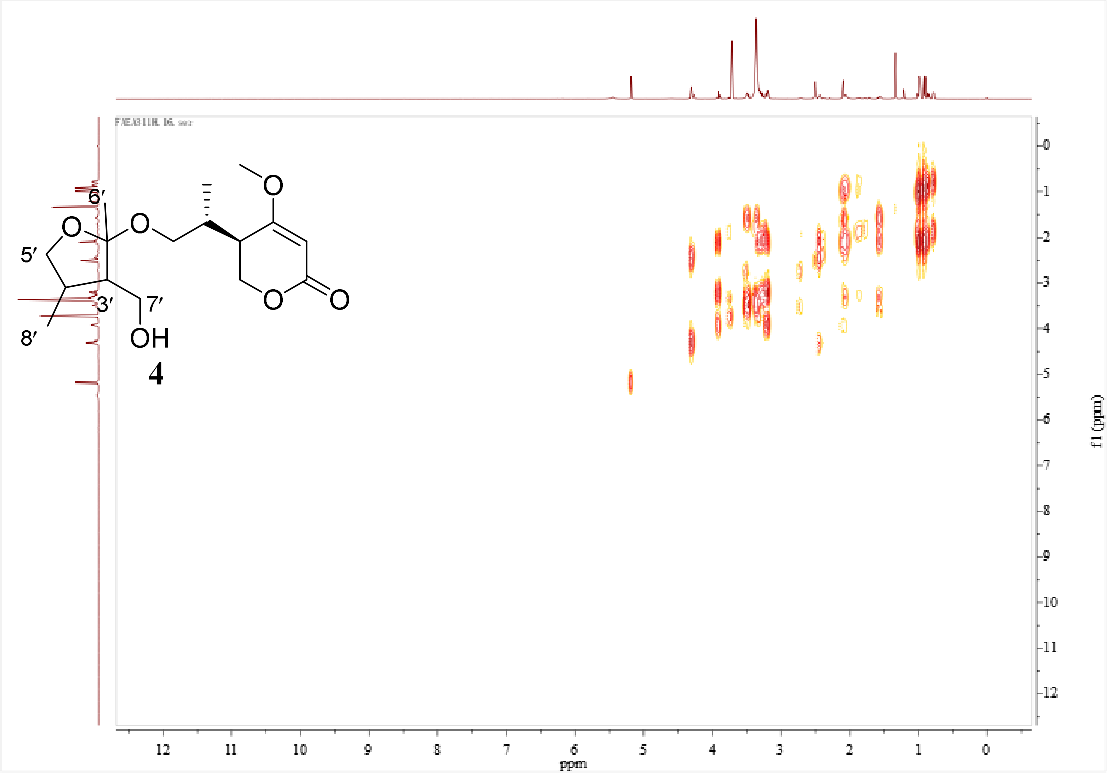

Figure S29. ^{1}H-^{1}H COSY spectrum of **4** in DMSO-d_6

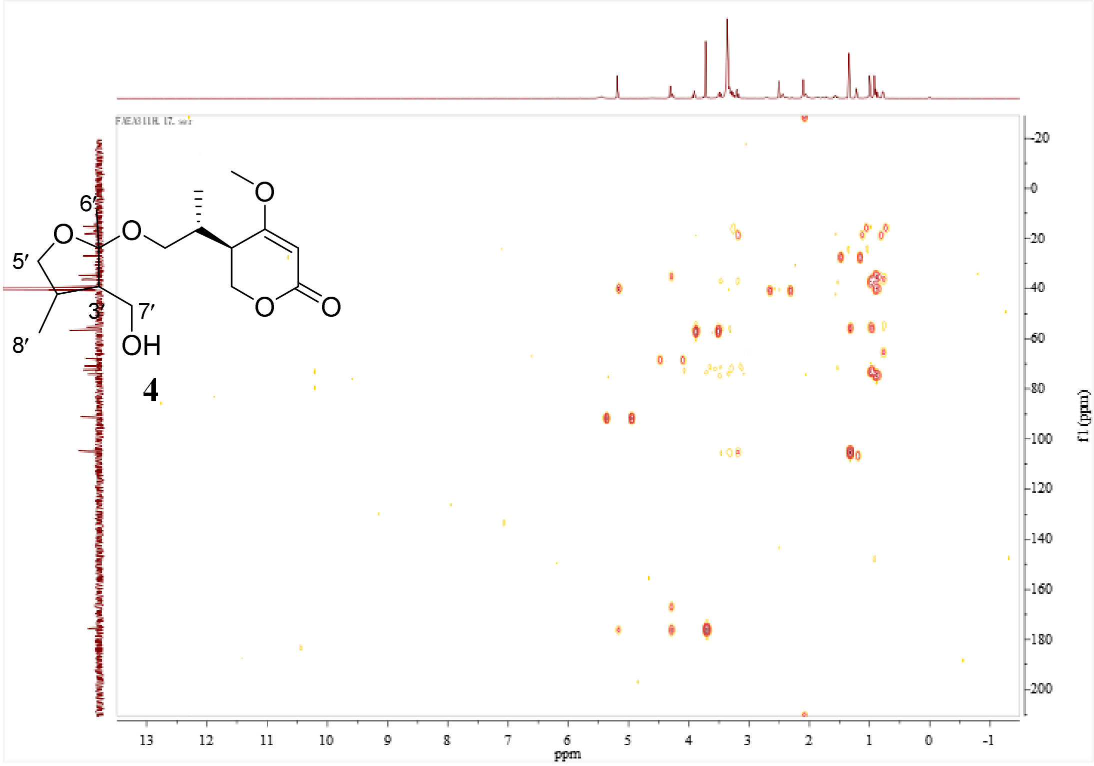

Figure S30. HMBC spectrum of **4** in DMSO-d_6

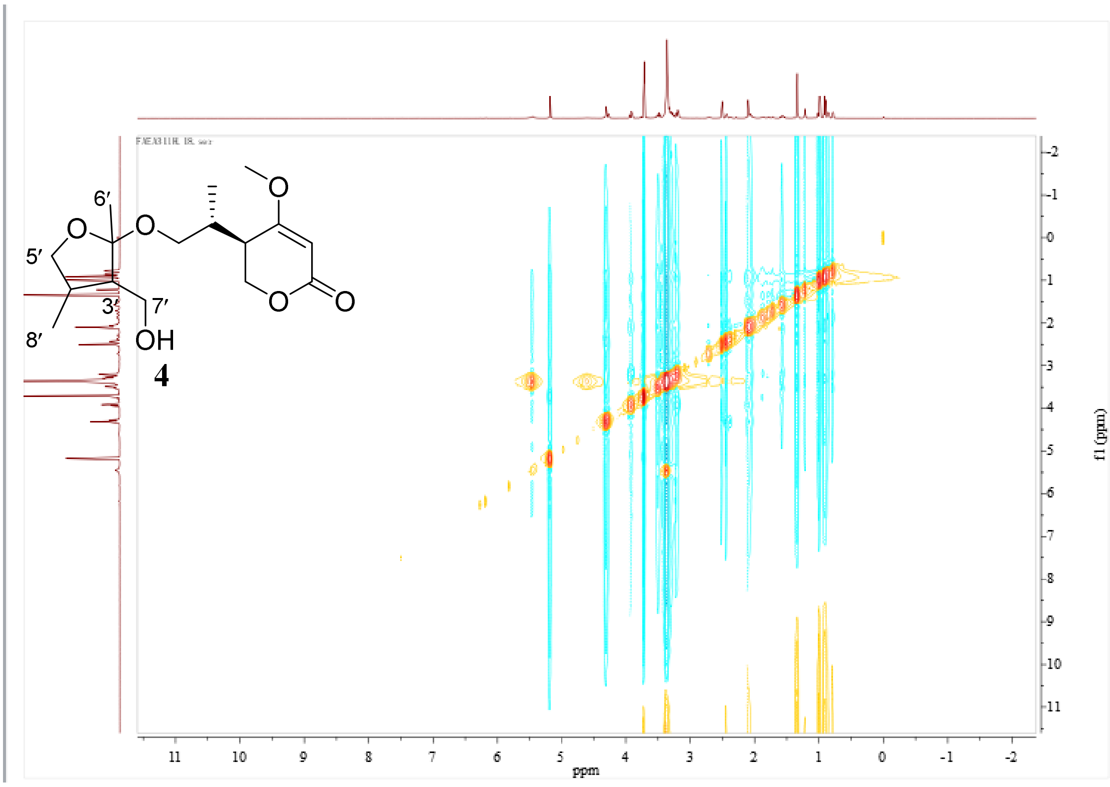

Figure S31. NOESY spectrum of **4** in DMSO-d_6

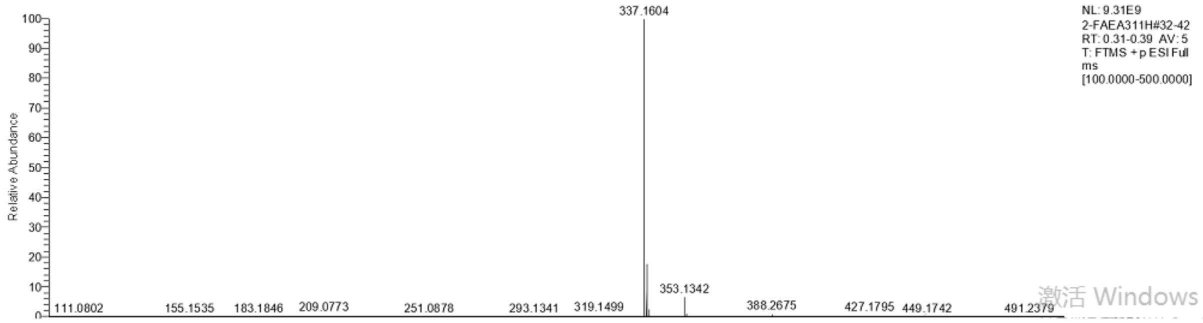

Figure S32. HRESIMS spectrum of **4**
